# Observation of Ultra‐High‐*Q* Resonators in the Ultrasound via Bound States in the Continuum

**DOI:** 10.1002/advs.202402917

**Published:** 2024-07-04

**Authors:** Mohamed Farhat, Younes Achaoui, Julio Andrés Iglesias Martínez, Mahmoud Addouche, Ying Wu, Abdelkrim Khelif

**Affiliations:** ^1^ Computer, Electrical, and Mathematical Sciences and Engineering Division King Abdullah University of Science and Technology (KAUST) Thuwal 23955‐6900 Saudi Arabia; ^2^ Institut FEMTO‐ST, CNRS UMR 6174 University Bourgogne Franche‐Comté 15B Avenue des Montboucons Besançon Cedex 25000 France; ^3^ Faculté des sciences Université Moulay Ismail Meknes bp 11201 Morocco; ^4^ Physical Science and Engineering (PSE) Division King Abdullah University of Science and Technology (KAUST) Thuwal 23955‐6900 Saudi Arabia; ^5^ College of Science and Engineering Hamad Bin Khalifa University Doha Qatar

**Keywords:** bound states in the continuum, Fabry–Perot resonators, high quality factors, metasurfaces, ultrasound, underwater acoustics

## Abstract

The confinement of waves in open systems represents a fundamental phenomenon extensively explored across various branches of wave physics. Recently, significant attention is directed toward bound states in the continuum (BIC), a class of modes that are trapped but do not decay in an otherwise unbounded continuum. Here, the theoretical investigation and experimental demonstration of the existence of quasi‐bound states in the continuum (QBIC) for ultrasonic waves are achieved by leveraging an elastic Fabry–Pérot metasurface resonator. Several intriguing properties of the ultrasound quasi‐bound states in the continuum that are robust to parameter scanning are unveiled, and experimental evidence of a remarkable *Q*‐factor of 350 at ≈1 MHz frequency, far exceeding the state‐of‐the‐art using a fully acoustic underwater system is presented. The findings contribute novel insights into the understanding of BIC for acoustic waves, offering a new paradigm for the design of efficient, ultra‐high *Q*‐factor ultrasound devices.

## Graphical abstract

Underwater ultrasound bound states in the continuum are theoretically investigated and experimentally demonstrated in a coupled elastic Fabry–Perot resonator system. The work presents a significant breakthrough in measuring an exceptionally high Q‐factor of 350 at 1 MHz, within a simple underwater device. This achievement is attributed to leveraging elastic BICs, where elastic metasurfaces composed of thin‐elastic silicon slabs with cut‐through slits are employed.

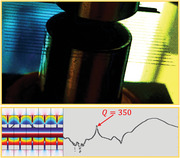

## 1 Introduction

The pursuit of acoustic high quality‐factor (*Q*‐factor, or simply *Q*) resonators and systems has been a sustained endeavor spanning several years, driven by the potential of unlocking numerous compelling applications in acoustic metamaterials and metasurfaces.^[^ ^1^, ^2^, ^3^ ^]^ Despite remarkable progress, various components, including acoustic sensors^[^ ^4^, ^5^ ^]^ for position and pressure, acoustic sources like sound ‘lasers’,^[^ ^5^ ^]^ and acoustic transducers including microphones and loudspeakers, alongside numerous other acoustic devices^[^ ^6^ ^]^ still face challenges in achieving efficiently high‐*Q* resonant characteristics. This challenge primarily stems from the scarcity of acoustic resonators exhibiting *Q*‐factors exceeding 100. To date, the *Q*‐factors of resonances in underwater experimental setups have been confined to the range of a few tens,^[^ ^4^, ^7^ ^]^ in stark contrast to the capabilities observed in optics, where *Q*‐factors can reach a few millions^[^ ^8^ ^]^ or even billions.^[^ ^9^ ^]^ The complexity further amplifies in the higher end of the sound spectrum, namely ultrasound, i.e., megahertz frequencies (MHz), and especially in aqueous environments where losses are prominent.

Underwater acoustic metamaterials^[^ ^10^ ^]^ hold great potential to revolutionize underwater communication and navigation, owing to the significant attenuation of electromagnetic waves in these environments, which makes their use virtually obsolete.^[^ ^11^, ^12^, ^13^ ^]^ Along these lines, a plethora of revolutionizing applications have been proposed, such as underwater perfect absorbers,^[^ ^14^ ^]^ cloaking devices,^[^ ^15^ ^]^ ultrasound vortex beams for enhanced emission,^[^ ^16^ ^]^ topological underwater structures.^[^ ^17^ ^]^ In the same vein, as ocean noise steadily rises due to human activities like cargo shipping, fishing, sonar usage, etc, it becomes increasingly urgent to understand the extent to which this noise alters the ocean's acoustic environment and affects marine ecosystems, such as coral reefs, i.e., by getting a clear picture of the ocean soundscape.^[^ ^18^ ^]^ And in this realm ultrasound underwater acoustics may play the central role.

Within the ultrasound spectrum, the *Q*‐factor of a resonator encounters various constraints, with viscous damping in water playing a significant role. The presence of viscosity causes energy dissipation within the resonator,^[^ ^19^, ^20^, ^21^ ^]^ leading to a reduction in the *Q*‐factor. This phenomenon becomes more pronounced at higher frequencies and in confined spaces such as narrow channels.^[^ ^22^ ^]^ Additionally, acoustic radiation introduces another constraint, especially in the MHz range, as the resonators emit acoustic energy into the surrounding medium, particularly in open (non‐Hermitian) environments.^[^ ^23^ ^]^ This emission contributes to energy loss, further reducing the *Q*‐factor. Material absorption, nonlinear effects, and temperature variations also negatively influence the confinement of the resonator. The combined impact of these factors poses significant challenges in achieving *Q*‐factor beyond 100 at MHz frequencies in water.^[^ ^24^ ^]^ Circumventing these challenges by leveraging new principle is thus crucial for unlocking the full potential of high‐*Q* resonators in various applications, from advanced acoustic sensors to cutting‐edge acoustic metamaterials.

Recently, a new class of acoustic open resonators emerged, presenting the potential for achieving ultra‐high‐*Q* factors^[^ ^25^, ^26^ ^]^ through the utilization of bound states in the continuum(BIC). However, the focus of these studies has been on airborne acoustics at lower frequencies in the range of a few kilohertz (kHz). When transitioning to ultrasounds in the MHz range in water, the currently attainable *Q*‐factors remain confined to the tens, primarily due to various influencing factors mentioned earlier.^[^ ^27^ ^]^

BIC exhibits a captivating phenomenon where wave modes are trapped in a specific region of space, even within an open system that permits energy to flow in and out.^[^ ^28^, ^29^, ^30^ ^]^ The emergence of BIC paved the way for the creation of resonant structures with high‐*Q* resonances.^[^ ^28^, ^29^, ^30^ ^]^ Originally discovered in quantum mechanics by von Neumann and Wigner, BIC results from the symmetry in the spatial distribution of the potential used to describe the wave equation, thereby creating a potential with localized eigen‐fields at zero energy.^[^ ^31^ ^]^ In quantum mechanics,^[^ ^32^ ^]^ a BIC refers to a state of a particle that is confined to a potential well, demonstrating a tendency to remain localized in one region. It arises when the corresponding potential possesses a high degree of symmetry, interfering with outgoing waves to trap the mode within the energy continuum.^[^ ^33^ ^]^ Consequently, this enables the mode to persist over an extended duration, despite its surrounding being an open system.^[^ ^34^ ^]^

The application of BIC extends beyond quantum mechanics and has been since then observed in various physical systems, including optics,^[^ ^35^, ^36^, ^37^, ^38^, ^39^, ^40^, ^41^ ^]^ guided systems,^[^ ^42^, ^43^ ^]^ chiral metamaterials,^[^ ^44^, ^45^, ^46^ ^]^ elasticity,^[^ ^47^, ^48^, ^49^ ^]^ and acoustics.^[^ ^23^, ^25^, ^26^, ^50^, ^51^, ^52^, ^53^, ^54^, ^55^, ^56^, ^57^, ^58^ ^]^ In particular, in acoustics, BIC can be created in resonant structures such as air cavities, narrow slits, and other systems that can partially confine sound waves or give rise to Fano^[^ ^41^ ^]^ resonances.^[^ ^59^, ^60^, ^61^, ^62^ ^]^ In general, an acoustic BIC may be attributed to two mechanisms: One involving the symmetry in the geometry of the resonant structure, known as symmetry‐protected BIC resulted from the destructive interference between leaking waves.^[^ ^50^, ^52^ ^]^ This mechanism is used to create ultra‐high *Q*‐factor resonances, leading to the development of devices such as filters, sensors, and resonant transducers.^[^ ^23^ ^]^ The other mechanism involves mode coupling between resonant modes in different regions of the structure, which results in coupling‐induced resonances. This can lead to either Fabry–Pérot BIC (FP‐BIC throughout this study) if the resonance frequencies of the cavities are equal^[^ ^28^, ^51^ ^]^ or Friedrich–Wintgen (FW‐BIC) if the frequencies are different.^[^ ^28^, ^56^ ^]^

Building upon this progress, our study aims to leverage the unique properties of BIC to create the highest *Q*‐factor underwater ultrasound resonator. We present both theoretical analysis and experimental observations demonstrating that a double elastic metasurface, composed of an array of slits in a silicon plate, and immersed in water, can support FP‐QBIC with an unprecedentedly high *Q*‐factor. Experiments confirm the existence of a *Q*‐factor on the order of 350, representing a substantial advancement beyond the current state‐of‐the‐art in ultrasound resonators in a water environment.

## 2 Results

**Figure 1:**
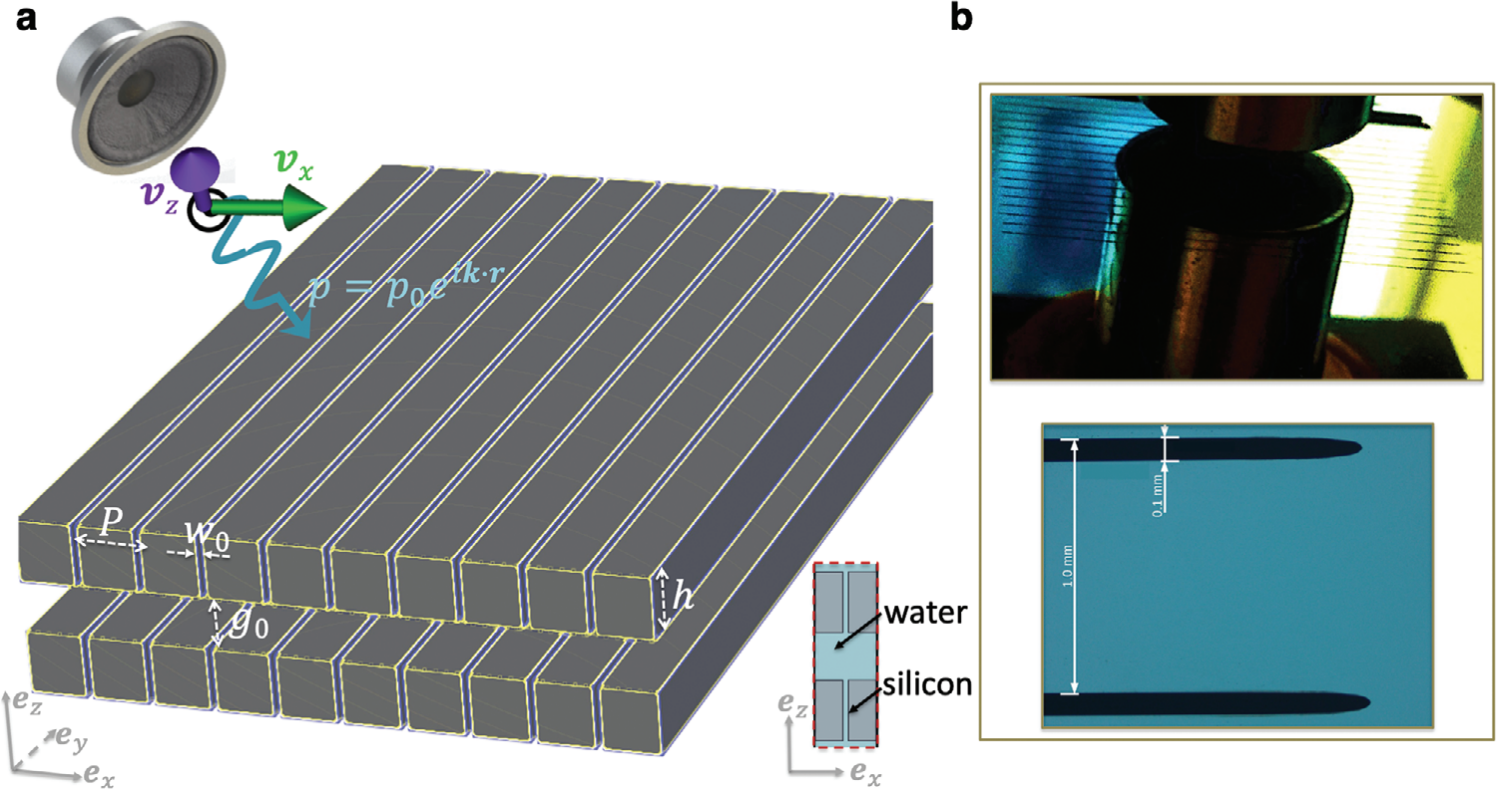
Scheme of the QBIC ultrasound metasurface. a) Ultrasound waves in the frequency range of 0.5–2 MHz are generated (schematically) via an ultrasound transducer (schematized here as a speaker) at an oblique incidence on a periodic metasurface embedded in aqueous environment (water), where the front view of the unit‐cell is depicted in the bottom inset, with the corresponding geometry and materials. b) Top panel: A photograph of the metasurface in front of the source. Bottom panel: Optical microscope image of a unit‐cell.

The metasurface under consideration is schematized in **Figure** **1**. It comprises two parallel silicon slabs, each has 1 mm thickness in the *z*‐direction, perforated with a periodic arrangement of thin slits aligned in the *x*‐direction and extending infinitely in the *y*‐direction (see inset of Figure 1 for the cross view). The silicon slabs are immersed in water. As a consequence, the slits are filled with water. Such configuration makes the problem numerically 2D. Each slab exhibits a periodicity of 1 mm. The slit, positioned at the center of the unit‐cell (UC) has a width of 0.1 mm. In fact, the position of the slit in the *x*‐direction is later demonstrated to have negligible influence for our purposes (Note S3, Supporting Information). The medium surrounding the UC shown in the inset of Figure 1 is water, which also fills the slit.

### 2.1 Single‐Layer Elastic Metasurfaces

**Figure 2:**
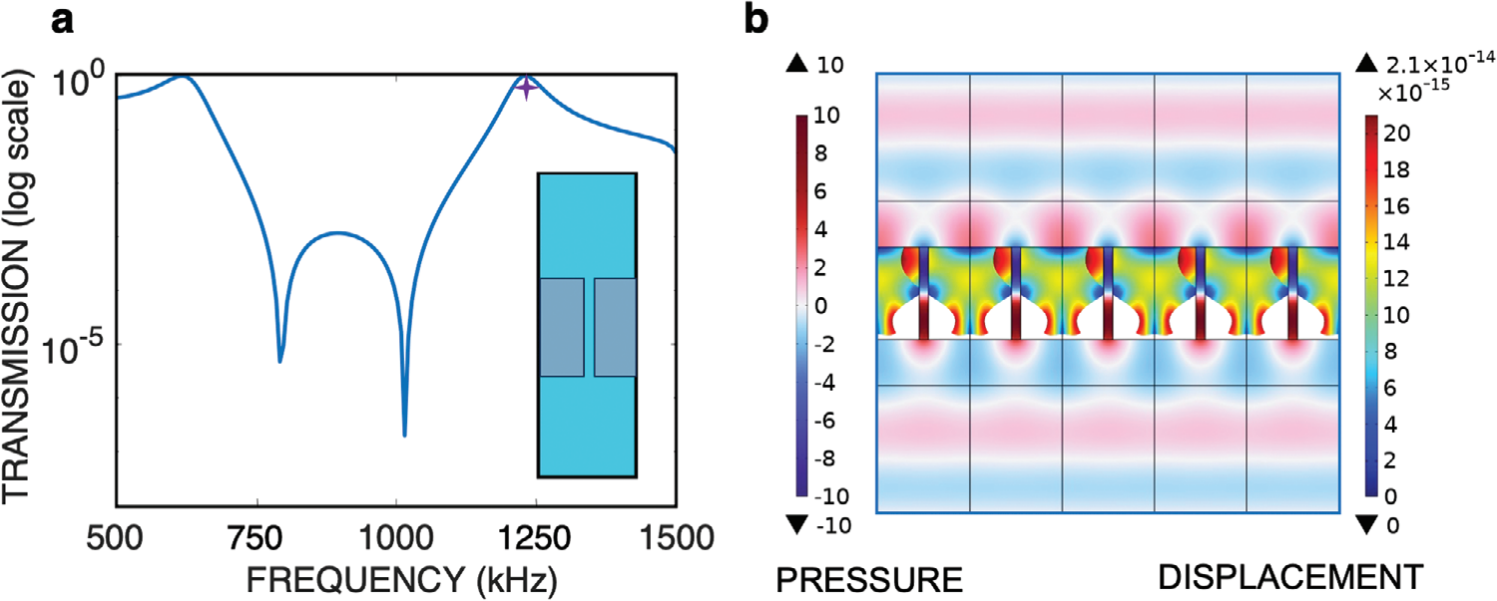
Transmission characteristics of the single‐layer elastic metasurface. a) Transmission in logarithmic scale of the single acoustic metasurface (shown in the inset). b) Real part of the pressure field (left color bar) and displacement amplitude (right color bar) at the frequency 1231.1 kHz, corresponding to the second peak, highlighted by a purple star in Figure 2a.

To gain a further understanding on the building blocks of our BIC device, we first focus on a structure that contains a single metasurface, i.e., a single layer of slitted‐silicon as shown in the inset of **Figure** **2**a. A somehow related structure was previously considered in ref. [63] for extraordinary sound transmission and also in ref. [64] where a finite‐length fluid channel between two elastic plates were studied. There, it was demonstrated the important role of the elastic eigenmodes of two plates acoustically coupled through a fluid channel in energy conservation, via sound re‐direction.^[^ ^64^ ^]^ We use the finite‐element software COMSOL Multiphysics^[^ ^65^ ^]^ to characterize the scattering response (transmission/reflection spectrum) of this metasurface (a single silicon slab) for ultrasound frequencies ranging from 500 to 1500 kHz. The propagation of sound waves in water is described via pressure acoustic module and that in silicon via solid mechanics module, as silicon supports both longitudinal and shear waves propagation (Note S1, Supporting Information).

Figure 2a presents the transmittance (|*t*|^2^) spectrum of this metasurface in logarithmic scale under normal incidence, exhibiting some regular FP resonances ≈600 and 1200 kHz and two dips resembling a *W*‐shape ≈830 and 1000 kHz. It should be emphasized that these resonances are broadband. For instance, the *Q*‐factor of a resonance is defined as *Q* = ω_0_/Δω, where ω_0_ is the resonance frequency and Δω is the full‐width‐at‐half‐maximum (FWHM) or alternatively and equivalently as *Q* = ω_0_/2Γ, with Γ being the imaginary part of the complex frequency ω = ω_0_ − *i*Γ that represents the radiative decay rate of the leaky mode. The resonances shown in Figure 2a have a low *Q*‐factor of ≈10. In particular, the resonance highlighted by a purple star has a near‐field of pressure, depicted in Figure 2b that is not perfectly localized within the metasurface, indicating high leakage or Γ ≈ ω_0_. The amplitude of pressure (its real part) and the displacement field |ℜ(**w**)| in the solid silicon layers are comparatively low, which is characteristics of an overdamped acoustic resonance.

Hence, this single metasurface cannot be used alone for the applications that we envision, e.g., sensing or acoustic lasing systems that require a *Q*‐factor of the order of a few hundreds, which is still lacking for underwater ultrasound waves.

### 2.2 Double Unit‐Cell Elastic Metasurfaces and Ultrasound QBIC

To address the challenges of low *Q*‐factors at ultrasound frequencies, where losses are prominent, we leverage the concept of BIC. This phenomenon is known to yield diverging *Q*‐factors for certain modes embedded in the continuum of radiating modes. Hence, BICs result in resonances that do not couple to the continuum of radiation owing to the destructive interference in our Fabry–Perot scenario or owing to symmetry‐protection in other BIC systems.

**Figure 3:**
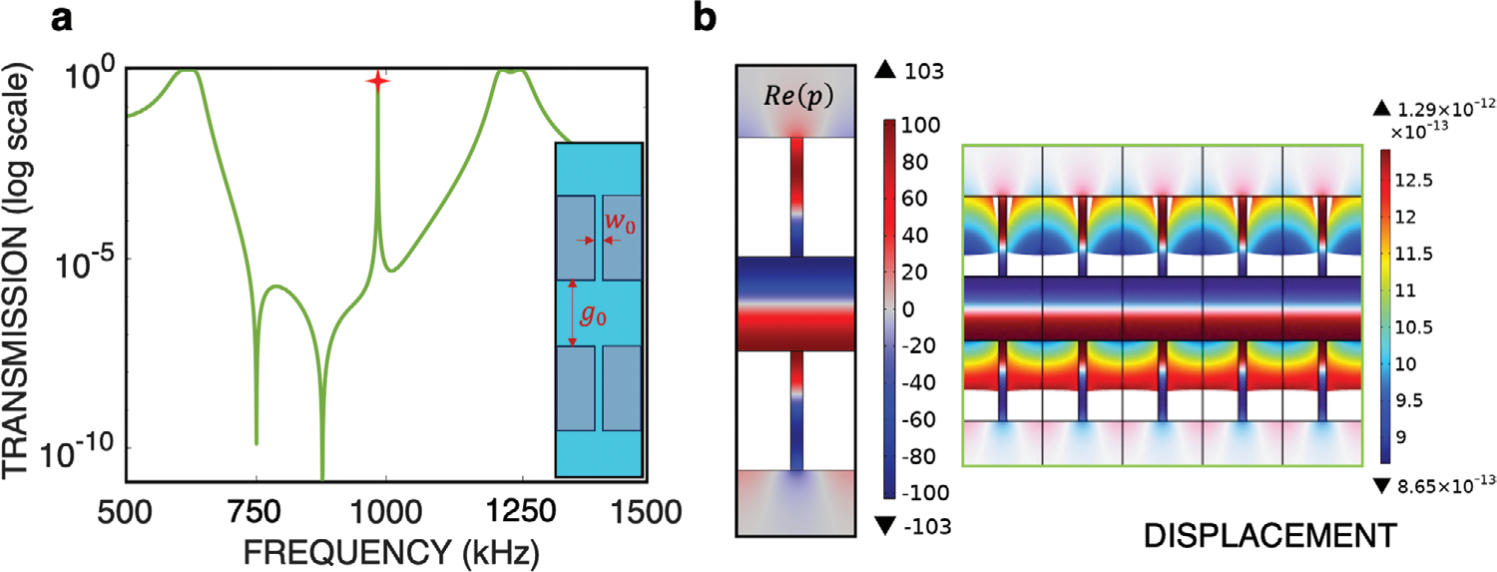
Transmission characteristics of the QBIC cavity. a) Transmission for the double‐layer metasurface (QBIC resonator, shown in the inset) in logarithmic scale. b) Real part of the total pressure field at the QBIC point (left panel), highlighted by a red star in Figure 3a and amplitude of the displacement field at the same frequency (right panel).

To exploit this effect, we utilize the same metasurface depicted in the inset of Figure 2a, by cascading two such metasurfaces, as illustrated in the inset of **Figure** **3**a. This configuration is designed to potentially trap acoustic radiation in the gap between the two metasurfaces, leading to the emergence of BIC under specific conditions. For instance, the BIC takes place when the gap thickness *g*_0_ is chosen in such a way that the accumulated phase in a single round‐trip equals *m* × 2π, with m∈N, where N denotes the set of natural numbers. This principle reflects the analogy with Fabry–Pérot cavities, typically composed of two mirrors. It is worthy to emphasize the importance of using a solid as the building block for the metasurface to allow the generation of BIC. If a hard wall is used instead, the QBIC disappears as shown in Note S2 (Supporting Information) (considering oblique incidence in these cases). This underscores the unique contribution of the solid‐based metasurface to create and sustain the QBIC in the ultrasound regime.

To gain insight of this FP‐BIC, we employ the temporal coupled‐mode theory (TCMT).^[^ ^66^, ^67^, ^68^ ^]^ We denote the amplitude of the resonances as a state vector Ψ = (ψ_1_, ψ_2_)^*T*^ that obeys the governing equation $\hat{H}\Psi =i\partial \Psi /\partial t$, with the Hamiltonian operator written as:

(1) $$\hat{H}=\left(\begin{array}{cc} \omega_{0} & \delta \\ \delta & \omega_{0} \end{array}\right)-i\Gamma \left(\begin{array}{cc} 1 & e^{i\varphi }\\ e^{i\varphi } & 1 \end{array}\right)\;.$$

Here, ω_0_ is the resonance frequency of the single metasurface, highlighted by the purple star in Figure 2a, which is the same for both metasurfaces. It is crucial to note that if the two metasurfaces possess distinct resonant frequencies, ω_1_ and ω_2_ for instance, the resulting BIC will be fundamentally different, i.e., a FW‐BIC.^[^ ^28^ ^]^ δ represents the near‐field coupling between the two metasurfaces, a parameter strongly dependent on the gap *g*_0_ as can be seen in the last sub‐section of Note S3 (Supporting Information), and with the frequency ω_1_ and the QBIC frequency, we have δ = 1.55 × 10^6^ rad s^−1^ (2π × 246.7 kHz). Γ = 1.98 × 10^5^ rad s^−1^ (2π × 31.5 kHz) is the radiative decay rate of the single metasurface and φ = *kg*_0_ is the phase‐shift between the resonators, where *k* denotes the acoustic (pressure) wavenumber. This system possesses two eigen‐solutions Ψ_±_, represented by the eigen‐values of Equation (1):

(2) $$\omega_{\pm }=\omega_{0}\pm \delta -i\Gamma \left(1\pm e^{i\varphi }\right)\;.$$

From Equation (2) it is clear that when φ = *m* × π, meaning the phase accumulation along one round‐trip is *m* × 2π, the eigen‐frequencies of the Hamiltonian simplify to ω_±_ = ω_0_ ± δ − *i*Γ(1 ± (− 1)^*m*^). Hence, one of the modes (Ψ_−_) exhibits a zero imaginary part, while the other (Ψ_+_) possesses an imaginary part of 2Γ, i.e., a radiative decay rate double that of the single metasurface. The occurrence of a purely‐real eigen‐frequency characterizes an authentic BIC.

Figure 3a illustrates the numerically calculated transmission of a double metasurface sharing identical parameters with those in Figure 2a, albeit featuring a gap of *g*_0_ = 0.8 mm (Referring to Note S3, Supporting Information for confirmation that the position of the slit in the *x*‐direction does not affect our results). In addition to the previously observed maxima and minima resembling a *W*‐shape with the single metasurface, a distinct ultra‐narrow peak emerges at *f*_0_ = 984.5 kHz in the double metasurface scenario. The distinguishing feature signaling the nature of this resonance as a QBIC is primarily its remarkably high *Q*‐factor, estimated here at ≈16 000, a number of three orders of magnitude higher than the *Q*‐factor associated with the single metasurface design. Furthermore, the observation of Figure 3b with both the real part of the pressure field (left panel) and the displacement field in elastic solid (right panel) shows the strong confinement of acoustic waves around the metasurface. The acoustic energy attains exceptionally high values in the gap and the slits, reaching ≈10^4^, and similarly the elastic displacement is two orders of magnitude higher compared to the single metasurface case. In addition, the QBIC frequency predicted by the simplified TCMT gives a value of 935 kHz, closely aligning (within a 4% error margin) with the observed frequency in Figure 3a, denoted by the red star. The slight discrepancy between these two values may be attributed to several factors, such as the metasurface sustaining both shear and longitudinal waves^[^ ^69^ ^]^ as well as to the presence of slits in the silicon metasurface. In fact, the mechanism of the FP‐BIC that we investigated, as explained in this section relies on destructive interference:^[^ ^26^, ^35^ ^]^ The wave accumulates a multiple of π phase in a single round‐trip. In this simple model, the gap size, i.e., *g*_0_, is used to evaluate the accumulation of the phase change represented by the term φ in Equation (2). But in reality, the existence of the slits and the solid‐fluid coupling may alter the true phase change.^[^ ^63^ ^]^ In addition, TCMT is a powerful yet simple analytical tool^[^ ^66^, ^67^ ^]^ that makes some assumptions and simplifications (as with many other analytical techniques) such as that the coupling between the various elements is weak, as well as linearity, conservation of energy, and time‐reversal invariance. While the three latter assumptions are guaranteed, the first one, i.e., the weak coupling approximation may indeed slightly limit the precision of the TCMT predictions. Having said all that, an discrepancy of 4%, though, can be considered by any means a good achievement.

### 2.3 Experimental Demonstration of FP‐BIC

**Figure 4:**
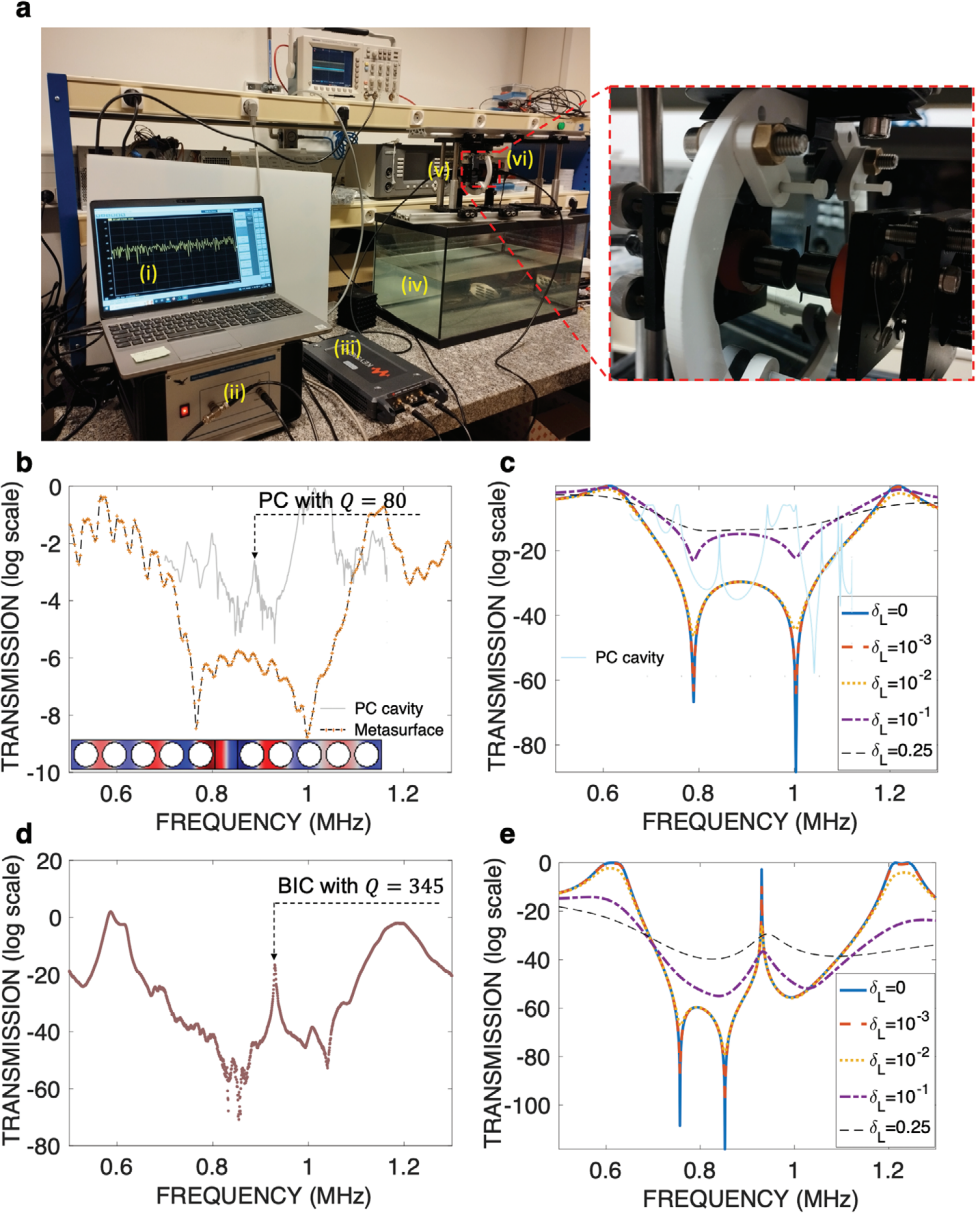
Experimental results. a) Experimental setup used to characterize the transmission/reflection spectrum of the metasurfaces in the ultrasound regime underwater: i) Ultrasound signal generation and data acquisition system; ii) Amplifier; iii) Vector network analyzer (VNA); iv) Water reservoir; v,vi) Basic mount with a rotating element (zoomed in in the inset). b) Experimental and c) numerical modeling (with various losses in the slits) of the transmission of the single silicon metasurface (log scale). The insets (transparent plots) give the transmission from a phononic cavity both numerically and experimentally using steel rods of diameter 2 mm, period of 2.22 mm and cavity size of 1.78 mm, schematized at the inset of Figure 4b (See Note S4, Supporting Information for details). d) Experimental and e) numerical modeling (with various loss in the slits) of the transmission of the double silicon metasurface (log scale). The measured *Q*‐factor of the BIC is 345.

We fabricate two metasurfaces made of silicon material with identical geometric properties as analyzed in previous sections. Our experimental setup, pictured in **Figure** **4**a, involves employing a dicing machine to create periodic perforations in two identical silicon wafers, as shown in both Figures 1b and 4a. Each silicon wafer possesses a thickness of 1 mm. The slits are positioned at intervals defined by a pitch *P* of 1 mm, each slit being 0.1 times the pitch in width, inducing *w*_0_ = 0.1 mm. This configuration covers an area of 33 × 40 mm^−2^ on the wafer's surface, equivalent to 33 periods and slits measuring 40 mm in length. To maintain a consistent cavity gap of *g*_0_ = 0.8 mm, a plastic ring is inserted between the two wafers.

Figure 4b,d depicts the measured transmission (log scale) of the single and double metasurface, respectively, for frequencies between 0.5 and 1.3 MHz. Excellent agreement is found between experimentally measured (Figure 4b,d) and numerically calculated results (Figure 4,e). Particularly, the resonant frequency found experimentally corresponds to a gap of 0.85 mm, falling within the experimental uncertainty related to fabrication and measurement errors (up to 4 %). The experimentally obtained *Q*‐factor, as illustrated in Figure 4d reaches 345 (refer to Note S4, Supporting Information for further experimental details). Even though this experimentally observed BIC is orders of magnitude lower than its lossless numerical counterpart (≈10^4^), it still offers a significant advancement for underwater ultrasound resonators. Moreover, to better fit the experimental measurements, we add some loss into the speed of sound inside the water slits, i.e., *c* = *c*_0_ × (1 + *i*δ_*L*_). By varying the value of δ_*L*_ we can see that the numerically simulated transmission approaches the experimentally measured results for both the single and double‐layer structures. A more complete investigation of the effect of loss as well as of other parameters, such as the temperature is given in Note S5 (Supporting Information). Resonators exhibiting similar *Q*‐factors in underwater ultrasound applications have not been reported so far. In the literature, the highest observed *Q*‐factors in this regime were achieved using phononic cavities, such as defects in 1D arrays of solid rods, for example, steel. To compare the Q‐factors of our BIC and the state‐of‐the‐art phononic cavity, we construct a phononic cavity using steel rods with a diameter of 2 mm, a period of 2.22 mm, comprising six rows, and a central gap of 1.78 mm, as shown in Figure 4b,c. We perform both numerical calculation and experimental measurement of transmission of such a resonator. The maximum *Q*‐factor that we can measure from it is ≈80. In addition, we analyzed in Note S5 (Supporting Information) the effect of using another material for the cavity, i.e., silicon, and found that in the phononic cavity case, steel permits the higher *Q*‐factors, which is opposite to the case of our metasurface.Hence, our FP‐BIC resonator not only demonstrates a substantially higher *Q*‐factor, providing valuable potential for future investigations in ultrasound sensors and acoustic imaging systems, but also differs significantly from the existing phononic crystal cavities.

Nevertheless, it is important to emphasize that the experimental parameter optimization is constrained by the source, which is regarded as an omnidirectional emitter radiating both in‐plane and out‐of‐plane (along the slit direction) of the metasurface. The out‐of‐plane radiation scenario lacks the same interference patterns as the in‐plane case and lacks the symmetry necessary to ensure the existence of QBIC. This means that the *Q*‐factor observed experimentally will suffer from these limitations and hence be reduced in comparison to the ideal scenario, which we confirm in Figure 4.

## 3 Discussion

### 3.1 Tunability and Dispersion of the QBIC

**Figure 5:**
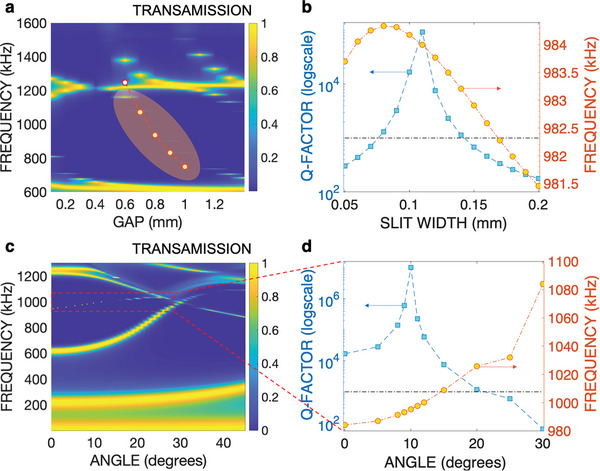
Effect of the gap and slit widths and dispersive properties. a) Contour plot of the transmission of the metasurface of the same parameters as in Figure 3a VS gap *g*_0_ and frequency. The white dotted curve depicts the TCMT predictions and the highlighted region shows the QBIC regime. b) *Q*‐factor for varying slit width *w*_0_ for a gap of 0.8 mm. The right *y*‐axis gives the frequency of the corresponding resonant QBIC. c) Dispersion curve of the BIC, i.e., versus incidence angle from 0 to 45°. The dashed red square denotes the location of the BIC, that is plotted in d) with its corresponding *Q*‐factor and resonance frequency (left and right *y*‐axis, respectively).

The frequency of the proposed FP‐QBIC is tunable. Beginning with the same metasurfaces as previously detailed in Figure 3a, we change the gap *g*_0_ between the two metasurfaces. The transmission data is plotted in **Figure** **5**a, with the *x*‐axis representing *g*_0_ in mm and the *y*‐axis representing frequency in kHz. The highlighted zone indicates the dependence of resonance frequency*f*_0_ on *g*_0_, agreeing well with analytical predictions from TCMT depicted by the white dotted curve in Figure 5a (additional details are provided in the first sub‐section of Note S3, Supporting Information). It is noteworthy that the QBIC disappears for smaller gaps and the *Q*‐factor reaches its optimum value for *g*_0_ ≈0.8 mm. Hence, we select *g*_0_ = 0.8 mm for experimental validation detailed in the next sub‐section. We also analyze the impact on the *Q*‐factor of altering the slit width *w*_0_ in Figure 5b. For small values of *w*_0_, such as ≈50 µm, the *Q*‐factor deteriorates. An optimal value is obtained for *w*_0_ = 0.11 mm, at which the *Q*‐factor reaches ≈10^5^. As *w*_0_ increases, the *Q*‐factor drops again, reinforcing that the most favorable BIC configuration corresponds to *w*_0_ ≈0.1*P*, i.e., 10% of the period of the metasurface. The corresponding change of the resonance frequency is also detailed in Figure 5b (additional details are provided in Note S3, Supporting Information).

It is also important to investigate the dispersive properties, specifically the angular dependence, of the QBIC mechanism. To accomplish this, we vary the incident angle of the incoming acoustic plane wave in the range 0–45° and plot the resulting transmission in a 2D format in Figure 5c. The regular broadband resonances, attributed to the FP interference, coexist with the QBIC (evidenced by a vanishing line‐width) as highlighted by the red‐dashed square. Figure 5d shows the dependence of the *Q*‐factors on incident angles, up to 30°. Notably, the *Q*‐factor demonstrates an increase from its value at normal incidence (≈10^4^) with ascending angles, peaking at ≈10^7^ for a 10° incidence, before decreasing. For angles exceeding 20°, the *Q*‐factor drops significantly below the threshold depicted by the dotted‐dashed‐black line corresponding to *Q* = 10^3^, indicating that the resonance can no longer be considered as a BIC. The notable increase in *Q*‐factor with angle and its optimum value ≈10° shown in Figure 5d (i.e., off‐Gamma point) that is reminiscent of accidental BIC^[^ ^28^, ^70^, ^71^ ^]^ may be in fact due to the excitation of both longitudinal and shear waves in silicon metasurfaces. In fact, we have shown the primordial role of elastic waves (both longitudinal and transverse) in the generation of our BIC effect (see Note S2, Supporting Information, where we have shown that using the same geometry but with a hard‐wall boundary condition instead of silicon does not lead to the ultra‐high *Q*‐factor.) This undoubtedly shows the role of the interplay between the shear and pressure waves, both excited at oblique incidence, in the silicon metasurface. This enables the elastic BIC and the subsequent high *Q*‐factor predicted theoretically and further observed experimentally. A further analysis is provided in Note S3 and Figure S10 (Supporting Information).

The resonance frequency of the BIC versus angle is illustrated by the right *y*‐axis of Figure 5d, and it consistently increases with the angle within the considered domain.

### 3.2 Mirror‐Induced QBIC

**Figure 6:**
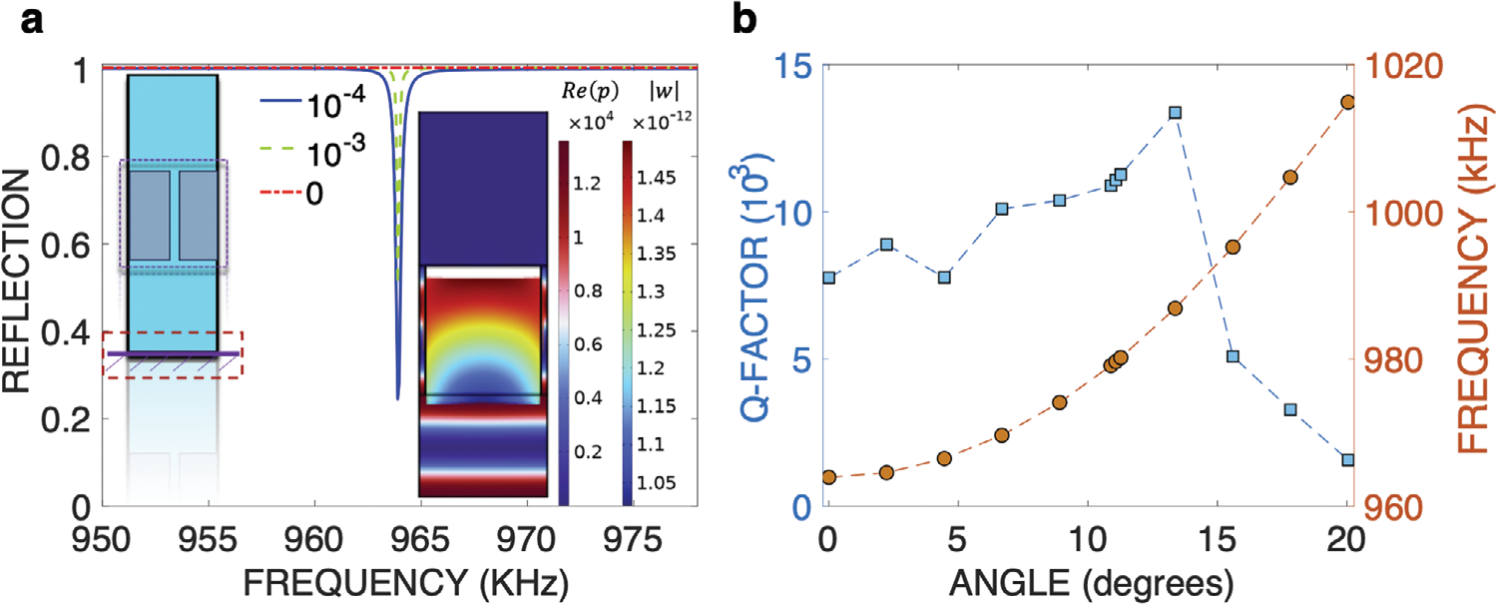
Single‐UC QBIC. a) Reflection spectrum for the single metasurface QBIC, schematized in the left inset, where a mirror is present at distance *g*_0_. The right inset depicts the real part of the pressure (*Re*(*p*)) and displacement (|**w**|) for that QBIC ≈965 kHz. b) *Q*‐factor (in units of 10^3^) of the obtained QBIC versus the angle of incidence (left axis: blue curve) and resonance frequency in kHz versus the angle of incidence (right axis: red curve).

We have demonstrated in Equation (1) that the formation of ultrasound QBIC necessitates two ‘elastic’ metasurfaces for both the FP and FW QBIC. However, a workaround to this limitation emerges by introducing a mirror (perfectly‐reflecting surface) beneath the single metasurface. We show that the FP‐QBIC can be observed under such conditions. When only a single metasurface is considered, the FP‐BIC disappears as the interference phenomenon is vital to QBIC formation (See Equation 2). In pressure acoustics, a perfect mirror is realized by enforcing sound hard‐wall boundary condition ($n\cdot \frac{1}{\rho }\nabla p$).^[^ ^72^ ^]^ The mirror, acting as the second resonator, ultimately leads to the QBIC, observed in **Figure** **6**a at a resonant frequency of 964 kHz, which is even closer to the TCMT predicted value. Importantly, to observe this QBIC, we need to add some loss in the narrow water channels; otherwise all the energy would be reflected (|*R*| = 1, in a lossless scenario, as depicted by the red dashed line in Figure 6a). The plots in Figure 6a showcase increasing losses (imaginary part in the speed of sound in water). The near‐field plots of both pressure and displacement are shown in the inset of Figure 6a, right panel, revealing a comparable enhancement to the double‐metasurface scenario. Of course, the quality of the mirror and its practical realization are pivotal to the QBIC properties and *Q*‐factor; these aspects will be further investigated in future studies.

In Figure 6b, the variation of both the *Q*‐factor and the resonance frequency of the QBIC in a single metasurface with a mirror when the angle of incidence of the wave varies between 0 and 20°. It is noticed that the *Q*‐factor increases with oblique incidence angle and reaches a maximum ≈14°, and then starts decreasing. This behavior bears some similarity to the case of the double‐metasurface QBIC shown in Figure 5d, albeit with a much smaller value of *Q*, potentially attributed to symmetry breaking in case of the hard‐wall mirror.

## 4 Conclusion

In summary, our work introduces a theoretical framework for ultra‐high *Q*‐factor ultrasound resonators designed for underwater acoustic applications. Experimental validation confirms the achievement of the targeted high‐*Q*, utilizing innovative design strategies leveraging the concept of QBIC with either two elastic metasurfaces or a single metasurface combined with a mirror. This approach exploits the destructive interference phenomenon to achieve unprecedented *Q*‐factors for the underwater ultrasound waves. The exceptional *Q*‐factors associated with FP resonances are intricately linked to the waveguide perturbation, offering a versatile means of attaining ultrahigh‐*Q* resonances without necessitating topological concepts, as exemplified in recent literature. What distinguishes our proposed approach is its potential for achieving ultrahigh *Q*‐factors while avoiding complicated processes that often introduce sample roughness and disorder. Our experimental demonstration, conducted at a wavelength of ≈1 MHz using a thin silicon‐slitted metasurface slab, yields remarkable *Q*‐factors of up to 350, rivaling those achieved through topological charge engineering. Furthermore, we achieve tunability of the resonant wavelength by varying the slit width, gap size, and/or angle of incidence.

Additionally, by generalizing our structure to 3D geometry, i.e., selecting distinct lattice constants along the *x*‐ and *y*‐axes, we can enhance the precision of realistic QBIC resonators. These findings represent a promising avenue for the development of ultrasonic and acoustic high‐*Q* devices, offering the potential to enhance performance in applications such as biosensors,^[^ ^73^ ^]^ reflectors,^[^ ^74^ ^]^ and filters through the utilization of ultra‐sharp spectral features.

## 5 Numerical and Experimental Methods

### Numerical Simulations

The numerical simulations were performed via the commercial software COMSOL Multiphysics.^[^ ^65^ ^]^ Sound waves propagating in water were modeled via the ‘Pressure Acoustics’, Frequency Domain (acpr)'. It was assumed that the density of water was 1000 kgm^−3^ and the speed of sound was 1498.6 m s^−1^. When fitting the experimental results, i.e., in Figure 4, the density and speed of sound were utilized from COMSOL Multiphysics library that vary with temperature. Some loss was also added in the speed of sound in the narrow slits, as discussed in Section 2.3. Elastic waves propagating in solid silicon were modeled via the ‘Solid Mechanics (solid)’. For silicon, a density of 2329 kgm^−3^, a Young's modulus of 170 GPa, and a Poisson's ratio of 0.28 were assumed. At the interface, multiphysics ‘Acoustic‐Structure Boundary’ conditions were used. In addition, to excite the structure, for any angle of incidence, background pressure field was used, wherean impinging plane acoustic wave of wave‐vector **k** = (*k*_*x*_, *k*_*y*_) was defined, and a given pressure amplitude and phase was taken as 1 and 0, respectively. The top and bottom domains were taken as perfectly matched layers (PMLs) to avoid domain reflections. The lateral boundary conditions were Floquet pseudo‐periodic with a **k**‐vector (*k*_*x*_, *k*_*y*_), both in the acpr and solid domains. The transmittance and reflectance were calculated by integration over a boundary on top and bottom of the metasurfaces, respectively.

### Metasurface Fabrication and Measurement

The silicon utilized in this experiment was of the <100 > crystallographic orientation, characterized by a mass density of 2329 kgm^−3^. Along the [100] direction, it exhibited longitudinal and transverse wave velocities of 8433 and 5843 m s^−1^, respectively. The experimental procedure relied on ultrasound transmission technique, wherein the sample was immersed in water and positioned between the generating and receiving transducers. These transducers, with a diameter of 15 mm, were broadband, centered at a frequency of 1 MHz frequency. A vector network analyzer (VNA) was employed to generate a wide‐ranging signal centered precisely at 1 MHz. This signal was then amplified to ensure a consistent power level for activating the transducer. The resulting pulse was received by a second transducer, linked to the second port of the VNA, which could perform a narrow‐band scan across a frequency range. At each frequency point, both the amplitude and phase of the signal were recorded. To establish a baseline, the transmission spectra were normalized by calculating the ratio between spectra obtained with and without the sample in place. Furthermore, an additional evaluation was conducted on a homogeneous membrane, classical FP resonator, as well as a phononic crystal to assess the impact of the quasi‐BIC confinement.

## Conflict of Interest

The authors declare no conflict of interest.

## Supporting information

Supporting Information

## Data Availability

The data that support the findings of this study are available from the corresponding author upon reasonable request.

## References

[1] M. Kadic , T. Bückmann , R. Schittny , M. Wegener , Rep. Prog. Phys. 2013, 76, 126501. 24190877 10.1088/0034-4885/76/12/126501

[2] G. Ma , P. Sheng , Sci. Adv. 2016, 2, e1501595. 26933692 10.1126/sciadv.1501595 PMC4771441

[3] S. A. Cummer , J. Christensen , A. Alù , Nat. Rev. Mater. 2016, 1, 1.

[4] W. Xu , S. Choi , J. Chae , Appl. Phys. Lett. 2010, 96, 5.

[5] M. Farhat , W. W. Ahmad , A. Khelif , K. N. Salama , Y. Wu , J. Appl. Phys. 2021, 129, 10.

[6] B. Assouar , B. Liang , Y. Wu , Y. Li , J.‐C. Cheng , Y. Jing , Nat. Rev. Mater. 2018, 3, 460.

[7] T. Biwa , Y. Ueda , H. Nomura , U. Mizutani , T. Yazaki , Phys. Rev. E 2005, 72, 026601. 10.1103/PhysRevE.72.026601 16196729

[8] X. Ji , S. Roberts , M. Corato‐Zanarella , M. Lipson , APL Photonics 2021, 6, 7.

[9] L. Wu , H. Wang , Q. Yang , Q.‐X. Ji , B. Shen , C. Bao , M. Gao , K. Vahala , Opt. Lett. 2020, 45, 5129. 32932469 10.1364/OL.394940

[10] E. Dong , P. Cao , J. Zhang , S. Zhang , N. X. Fang , Y. Zhang , Natl. Sci. Rev. 2023, 10, nwac246. 37181091 10.1093/nsr/nwac246 PMC10171648

[11] A. I. Al‐Shamma'a , A. Shaw , S. Saman , IEEE Trans. Ant. Prop. 2004, 52, 2843.

[12] X. Che , I. Wells , G. Dickers , P. Kear , X. Gong , IEEE Comm. Mag. 2010, 48, 143.

[13] S. Jiang , S. Georgakopoulos , J. Electr. Anal. Appl. 2011, 3, 5906.

[14] S. Qu , N. Gao , A. Tinel , B. Morvan , V. Romero‐García , J.‐P. Groby , P. Sheng , Sci. Adv. 2022, 8, eabm4206. 35584217 10.1126/sciadv.abm4206 PMC9116603

[15] S. Zhang , C. Xia , N. Fang , Phys. Rev. Lett. 2011, 106, 024301. 21405230 10.1103/PhysRevLett.106.024301

[16] X. Jiang , J. Zhao , S.‐l. Liu , B. Liang , X.‐Y. Zou , J. Yang , C.‐W. Qiu , J.‐C. Cheng , Appl. Phys. Lett. 2016, 108, 20.

[17] Y. Shen , C. Qiu , X. Cai , L. Ye , J. Lu , M. Ke , Z. Liu , Appl. Phys. Lett. 2019, 114, 2.

[18] C. M. Duarte , L. Chapuis , S. P. Collin , D. P. Costa , R. P. Devassy , V. M. Eguiluz , C. Erbe , T. A. Gordon , B. S. Halpern , H. R. Harding , M. N. Havlik , M. Meekan , N. D. Merchant , J. L. Miksis‐Olds , M. Parsons , M. Predragovic , A. N. Radford , C. A. Radford , S. D. Simpson , H. Slabbekoorn , E. Staaterman , I. C. Van Opzeeland , J. Winderen , X. Zhang , F. Juanes , Science 2021, 371, eaba4658. 33542110 10.1126/science.aba4658

[19] L. Korson , W. Drost‐Hansen , F. J. Millero , J. Phys. Chem. 1969, 73, 34.

[20] D. Homentcovschi , R. N. Miles , Wave Motion 2008, 45, 191. 19122753 10.1016/j.wavemoti.2007.05.006 PMC2441451

[21] P. M. Morse , K. U. Ingard , Theoretical acoustics, Princeton University Press, New Jersey, 1986.

[22] G. Ward , R. Lovelock , A. Murray , A. P. Hibbins , J. R. Sambles , J. Smith , Phys. Rev. Lett. 2015, 115, 044302. 26252688 10.1103/PhysRevLett.115.044302

[23] L. Huang , S. Huang , C. Shen , S. Yves , A. S. Pilipchuk , X. Ni , S. Kim , Y. K. Chiang , D. A. Powell , J. Zhu , Y. Cheng , Y. Li , A. F. Sadreev , A. Alú , A. E. Miroshnichenko , Nat. Rev. Phys. 2023, 6, 11.

[24] F. Pop , B. Herrera , M. Rinaldi , Nat. Commun. 2022, 13, 1782. 35379794 10.1038/s41467-022-29355-9 PMC8979945

[25] L. Huang , Y. K. Chiang , S. Huang , C. Shen , F. Deng , Y. Cheng , B. Jia , Y. Li , D. A. Powell , A. E. Miroshnichenko , Nat. Commun. 2021, 12, 4819. 34376653 10.1038/s41467-021-25130-4 PMC8355331

[26] L. Huang , B. Jia , A. S. Pilipchuk , Y. Chiang , S. Huang , J. Li , C. Shen , E. N. Bulgakov , F. Deng , D. A. Powell , S. A. Cummer , Y. Li , A. F. Sadreev , A. E. Miroshnichenko , Phys. Rev. Appl. 2022, 18, 054021.

[27] G. Hornig , K. Scheuer , E. Dew , R. Zemp , R. DeCorby , Opt. Express 2022, 30, 33083. 36242356 10.1364/OE.463588

[28] C. W. Hsu , B. Zhen , A. D. Stone , J. D. Joannopoulos , M. Soljačić , Nat. Rev. Mater. 2016, 1, 1.

[29] S. Joseph , S. Pandey , S. Sarkar , J. Joseph , Nanophotonics 2021, 10, 4175.

[30] K. Koshelev , A. Bogdanov , Y. Kivshar , A. Bagdanov , Opt. Photonics News 2020, 31, 38.

[31] J. von Neumann , E. P. Wigner , The Collected Works of Eugene Paul Wigner: Part A: The Scientific Papers, Springer, New Jersey, 1993, pp. 291–293.

[32] L. D. Landau , E. M. Lifshitz , Quantum mechanics: non‐relativistic theory, vol. 3, Elsevier, Netherlands, 2013.

[33] F. H. Stillinger , D. R. Herrick , Phys. Rev. A 1975, 11, 446.

[34] M. Le Bellac , Quantum physics, Cambridge University Press, Cambridge, 2011.

[35] C. W. Hsu , B. Zhen , J. Lee , S.‐L. Chua , S. G. Johnson , J. D. Joannopoulos , M. Soljačić , Nature 2013, 499, 188. 23846657 10.1038/nature12289

[36] D. Marinica , A. Borisov , S. Shabanov , Phys. Rev. Lett. 2008, 100, 183902. 18518374 10.1103/PhysRevLett.100.183902

[37] E. N. Bulgakov , A. F. Sadreev , Phys. Rev. B 2008, 78, 075105.

[38] Y. Plotnik , O. Peleg , F. Dreisow , M. Heinrich , S. Nolte , A. Szameit , M. Segev , Phys. Rev. Lett. 2011, 107, 183901. 22107630 10.1103/PhysRevLett.107.183901

[39] F. Monticone , A. Alu , Phys. Rev. Lett. 2014, 112, 213903.

[40] P. Pankin , B.‐R. Wu , J.‐H. Yang , K.‐P. Chen , I. Timofeev , A. Sadreev , Commun. Phys. 2020, 3, 91.

[41] T. C. Tan , E. Plum , R. Singh , Adv. Opt. Mater. 2020, 8, 1901572.

[42] S. Han , M. V. Rybin , P. Pitchappa , Y. K. Srivastava , Y. S. Kivshar , R. Singh , Adv. Opt. Mater. 2020, 8, 1900959.

[43] W. Wang , Y. K. Srivastava , T. C. Tan , Z. Wang , R. Singh , Nat. Commun. 2023, 14, 2811. 37198151 10.1038/s41467-023-38367-y PMC10192215

[44] A. Overvig , N. Yu , A. Alù , Phys. Rev. Lett. 2021, 126, 073001. 33666456 10.1103/PhysRevLett.126.073001

[45] Z. Zhou , B. Jia , N. Wang , X. Wang , Y. Li , Phys. Rev. Lett. 2023, 130, 116101. 37001097 10.1103/PhysRevLett.130.116101

[46] Y. Chen , H. Deng , X. Sha , W. Chen , R. Wang , Y.‐H. Chen , D. Wu , J. Chu , Y. S. Kivshar , S. Xiao , C.‐W. Qiu , Nature 2023, 613, 474. 36653568 10.1038/s41586-022-05467-6

[47] D. Lee , J. Park , S. Kim , J. Mun , J. Kim , X. Piao , N. Park , J. Rho , Extreme Mech. Lett. 2023, 61, 101965.

[48] M. Martí‐Sabaté , B. Djafari‐Rouhani , D. Torrent , Phys. Rev. Res. 2023, 5, 013131.

[49] M. Martí‐Sabaté , J. Li , B. Djafari‐Rouhani , S. A. Cummer , D. Torrent , Commun. Phys. 2024, 7, 122.

[50] A. Lyapina , D. Maksimov , A. Pilipchuk , A. Sadreev , J. Fluid Mech. 2015, 780, 370.

[51] M. Amrani , I. Quotane , C. Ghouila‐Houri , L. Krutyansky , B. Piwakowski , P. Pernod , A. Talbi , B. Djafari‐Rouhani , C. Ghouila‐Houri , E. Houssaine , E. Boudouti , Phys. Rev. Appl. 2021, 15, 054046.

[52] L. Huang , B. Jia , Y. K. Chiang , S. Huang , C. Shen , F. Deng , T. Yang , D. A. Powell , Y. Li , A. E. Miroshnichenko , Adv. Sci. 2022, 9, 2200257. 10.1002/advs.202200257 PMC9284153 35561061

[53] I. Deriy , I. Toftul , M. Petrov , A. Bogdanov , Phys. Rev. Lett. 2022, 128, 084301. 35275659 10.1103/PhysRevLett.128.084301

[54] A. Sadreev , E. Bulgakov , A. Pilipchuk , A. Miroshnichenko , L. Huang , Phys. Rev. B 2022, 106, 085404.

[55] Y. Chen , K. Wang , M. Kadic , S. Guenneau , C. Wang , M. Wegener , Commun. Phys. 2023, 6, 75.

[56] S. Liu , S. Huang , Z. Zhou , P. Qian , B. Jia , H. Ding , N. Wang , Y. Li , J. Chen , Phys. Rev. Appl. 2023, 20, 044075.

[57] F. Kronowetter , M. Maeder , Y. K. Chiang , L. Huang , J. D. Schmid , S. Oberst , D. A. Powell , S. Marburg , Nat. Commun. 2023, 14, 6847. 37891166 10.1038/s41467-023-42621-8 PMC10611717

[58] B. Jia , L. Huang , A. S. Pilipchuk , S. Huang , C. Shen , A. F. Sadreev , Y. Li , A. E. Miroshnichenko , Phys. Rev. Appl. 2023, 19, 054001.

[59] M. Amin , A. Elayouch , M. Farhat , M. Addouche , A. Khelif , H. Bagcı , J. Appl. Phys. 2015, 118, 164901.

[60] M. Amin , O. Siddiqui , M. Farhat , A. Khelif , J. Appl. Phys. 2018, 123, 144502.

[61] M. Amin , O. Siddiqui , M. Farhat , J. Lightwave Technol. 2021, 39, 7869.

[62] A. A. Bogdanov , K. L. Koshelev , P. V. Kapitanova , M. V. Rybin , S. A. Gladyshev , Z. F. Sadrieva , K. B. Samusev , Y. S. Kivshar , M. F. Limonov , Adv. Photonics 2019, 1, 016001.

[63] M. H. Lu , X. K. Liu , L. Feng , J. Li , C. P. Huang , Y. F. Chen , Y. Y. Zhu , S. N. Zhu , N. B. Ming , Phys. Rev. Lett. 2007, 99, 174301. 17995334 10.1103/PhysRevLett.99.174301

[64] A. Bozhko , V. M. García‐Chocano , J. Sánchez‐Dehesa , A. Krokhin , Phys. Rev. B 2015, 91, 094303.

[65] COMSOL Multiphysics, V5.6 (build: 280), https://www.comsol.com (accessed: October 2022).

[66] S. Fan , W. Suh , J. D. Joannopoulos , JOSA A 2003, 20, 569. 12630843 10.1364/josaa.20.000569

[67] L. Verslegers , Z. Yu , P. B. Catrysse , S. Fan , JOSA B 2010, 27, 1947.

[68] A. Overvig , S. A. Mann , A. Alù , *arXiv preprint arXiv:2307.01186* 2023, 85.

[69] K. F. Graff , Wave motion in elastic solids, Courier Corporation, Dover Publications Inc., New York, 2012.

[70] M. Kang , S. Zhang , M. Xiao , H. Xu , Phys. Rev. Lett. 2021, 126, 117402. 33798377 10.1103/PhysRevLett.126.117402

[71] B. Zhen , C. W. Hsu , L. Lu , A. D. Stone , M. Soljačić , Phys. Rev. Lett. 2014, 113, 257401. 25554906 10.1103/PhysRevLett.113.257401

[72] M. Farhat , S. Guenneau , A. Alù , Y. Wu , Phys. Rev. B 2020, 101, 174111.

[73] H. Zhang , T. Wang , J. Tian , J. Sun , S. Li , I. De Leon , R. P. Zaccaria , L. Peng , F. Gao , X. Lin , H. Chen , G. Wang , Nanophotonics 2021, 11, 297. 10.1515/nanoph-2021-0368 PMC11502010 39633879

[74] M. Luo , F. Wu , Phys. Rev. A 2022, 106, 063514.

