# Supplementary material for: Observation of Ultra‐High‐Q Resonators in the Ultrasound via Bound States in the Continuum
Source: Adv Sci (Weinh). 2024 Jul 4;11(33):2402917. doi: 10.1002/advs.202402917 (PMC11434233; doi:10.1002/advs.202402917)
Supplement: Supplementary file 1 — Supporting Information [file ADVS-11-2402917-s001.pdf]

ADVANCED SCIENCE
Open Access

Supporting Information

for *Adv. Sci.*, DOI 10.1002/advs.202402917

Observation of Ultra-High-*Q* Resonators in the Ultrasound via Bound States in the Continuum

Mohamed Farhat, Younes Achaoui, Julio Andrés Iglesias Martínez, Mahmoud Addouche, Ying Wu and Abdelkrim Khelif**

Supporting Information

Observation of Ultra-High-Q Resonators in the Ultrasound via Bound States in the Continuum

Mohamed Farhat,[1] Younes Achaoui,[2,3] Julio A. Iglesias Martínez,[2]
Mahmoud Addouche,[2] Ying Wu,[1,4,*] and Abdelkrim Khelif[2,5,†]

[1]*Computer, Electrical, and Mathematical Sciences and Engineering Division,*
King Abdullah University of Science and Technology (KAUST), Thuwal 23955-6900, Saudi Arabia

[2]*Institut FEMTO-ST, CNRS UMR 6174,*
University Bourgogne Franche-Comté,
15B Avenue des Montboucons, 25000 Besançon Cedex, France

[3]*Faculté des sciences, Université Moulay Ismail, Meknes, Morocco*

[4]*Physical Science and Engineering (PSE) Division,*
King Abdullah University of Science and Technology
(KAUST), Thuwal 23955-6900, Saudi Arabia

[5]*College of Science and Engineering,*
Hamad Bin Khalifa University, Doha, Qatar

(Dated: June 2, 2024)

Abstract

The Supporting Information includes:

Supplementary notes 1-6

Supplementary figures 1-15

Supplementary references

[*] ying.wu@kaust.edu.sa

[†] abdelkrim.khelif@femto-st.fr

NOTE 1: ROLE OF THE ENVIRONMENT ON THE BIC CREATION

We have seen in the main manuscript (abbreviated in the following as MS) that when silicon metasurfaces are embedded in water, the QBIC can take place. For instance, silicon in water must obey the full elasticity treatment, i.e., the presence of both pressure and shear waves. For instance, the general equation governing the scattering of elastic waves in solids is [1]

$$(\lambda+2\mu)\nabla\zeta-\mu\nabla\times(\mathbf{v}_s)=\rho\frac{\partial^2\mathbf{u}}{\partial t^2}\,, \tag{1}$$

with $\zeta=\nabla\cdot\mathbf{u}$ and $\mathbf{v}_s=\nabla\times\mathbf{u}$. From this equation, the dynamics of ζ and $\mathbf{v}_s$ are governed by

$$\Delta\zeta=\frac{\rho}{\lambda+2\mu}\frac{\partial^2\zeta}{\partial t^2}\,, \tag{2}$$

and

$$\Delta(\mathbf{v}_s)=\frac{\rho}{\mu}\frac{\partial^2(\mathbf{v}_s)}{\partial t^2}\,, \tag{3}$$

where we can define the pressure (longitudinal) and shear velocity as

$$c_p=\sqrt{\frac{\lambda+2\mu}{\rho}}\ \text{ and }\ c_s=\sqrt{\frac{\mu}{\rho}}\,. \tag{4}$$

Here, λ and μ are the Lamé parameters. These parameters are related to the more familiar ones: Young's modulus E, Poisson's ratio ν via the well-known conversion

$$\lambda=\frac{E\nu}{(1+\nu)(1-2\nu)}\ \text{ and }\ \mu=\frac{E}{2(1+\nu)}\,. \tag{5}$$

This permits the definition of the bulk modulus κ and shear modulus G via

$$c_p=\sqrt{\frac{\kappa}{\rho}}\ \text{ and }\ c_s=\sqrt{\frac{G}{\rho}}\,. \tag{6}$$

where

$$\kappa=\frac{1-\nu}{(1+\nu)(1-2\nu)}\times E\ \text{ and }\ G=\frac{1}{2(1+\nu)}\times E\,. \tag{7}$$

For silicon used throughout the MS, we have $E=170$ GPa, $\nu=0.28$, and $\rho=2329$ kg/m^3, leading to $c_p=9658$ m/s and $c_s=5335$ m/s.

When silicon is embedded inside water, we can compute the acoustic impedance, both pressure and shear, i.e., $\eta_p=\rho c_p$ and $\eta_s=\rho c_s$, and normalize them by $\eta_{\text{water}}=\rho_w c_w$. In this work, unless otherwise stated (i.e., everywhere in this study, except in Figure 4 of the MS, where we

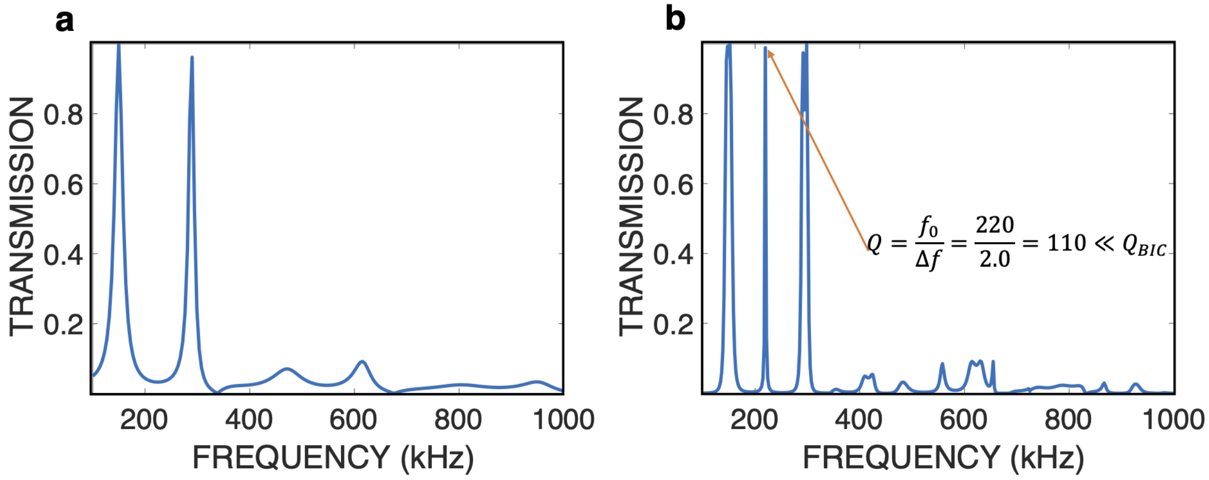

FIG. S1. **Role of the environment on the BIC. a** Transmittance for a single metasurface embedded in air. **b** Transmittance for the double metasurface supporting BIC, in the main manuscript, for a broad frequency range. The inset shows the maximum Q-factor obtained in this situation.

use a density and speed of sound varying with temperature) we assume $\rho_w = 1000$ kg/m^3 and $c_w = 1498.6$ m/s. We obtain thus $\overline{\eta}_s = \eta_s/\eta_w = 8.4$ and $\overline{\eta}_p = \eta_p/\eta_w = 15.2$. This means that both waves should be considered.

To verify the importance of both P and S waves to the QBIC observed in our MS, we reproduce the same results of Figs. 2(a) and 2(b) by replacing the host medium from water to air. The results of transmittance are depicted in Figure S1(a) and S1(b), for the single and double metasurface, respectively. For the single metasurface, as before, no QBIC can be observed, as confirmed in Figure S1(a). Yet, for the double metasurface, a mode appears between the broad resonances at around 210 kHz. Nevertheless, the Q-factor of this mode is a mere $110 \ll Q_{\mathrm{QBIC}}$. This showcases that a full elastic meta-atom is required for the emergence of our QBIC in the main MS. For instance, in air, $\overline{\eta}_p = 5.35 \times 10^4$ and $\overline{\eta}_s = 2.95 \times 10^4$ meaning that the silicon meta-atom behaves literally as a hard-wall boundary (i.e., $\mathbf{n} \cdot \nabla p = 0$).

NOTE 2: ROLE OF THE META-ATOM CONSTITUTING MATERIAL

Hard-wall material

The previous note shows the importance of having a low impedance mismatch between the meta-atom and the environment. To further investigate the role of the metasurface material, we keep

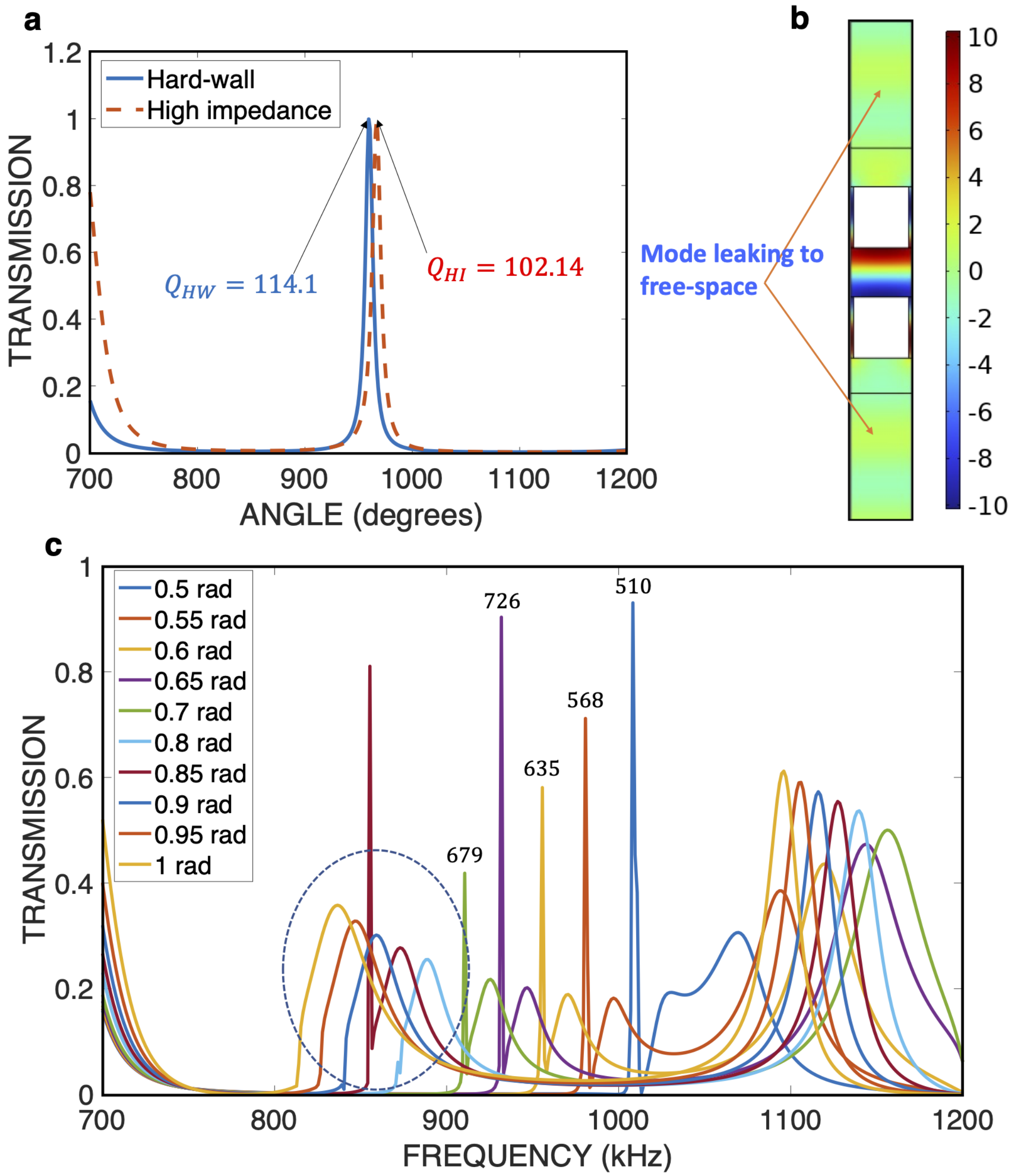

FIG. S2. **Hard-wall material unit-cell.** **a** Transmittance of the double metasurface (same geometry as the main manuscript) made of hard wall-material (blue continuous line) or very-high impedance (red-dashed line). The Q-factor obtained is around $10^2 \ll Q_{\text{QBIC}}$. **b** Real part of the pressure field around the resonance frequency of 959.4 kHz, which is of FP nature. **c** Oblique incidence for the hard-wall double metasurface for incident angles θ ranging from 0.5 to 1 rad. The Q-factors increase to a few hundreds but are still very small compared to the QBIC.

the geometry as before (i.e., the same as in Figure 2(b) of the MS) and we replace silicon by a hard-wall boundary condition and give the transmittance in Figure S2(a), blue line. The resonance

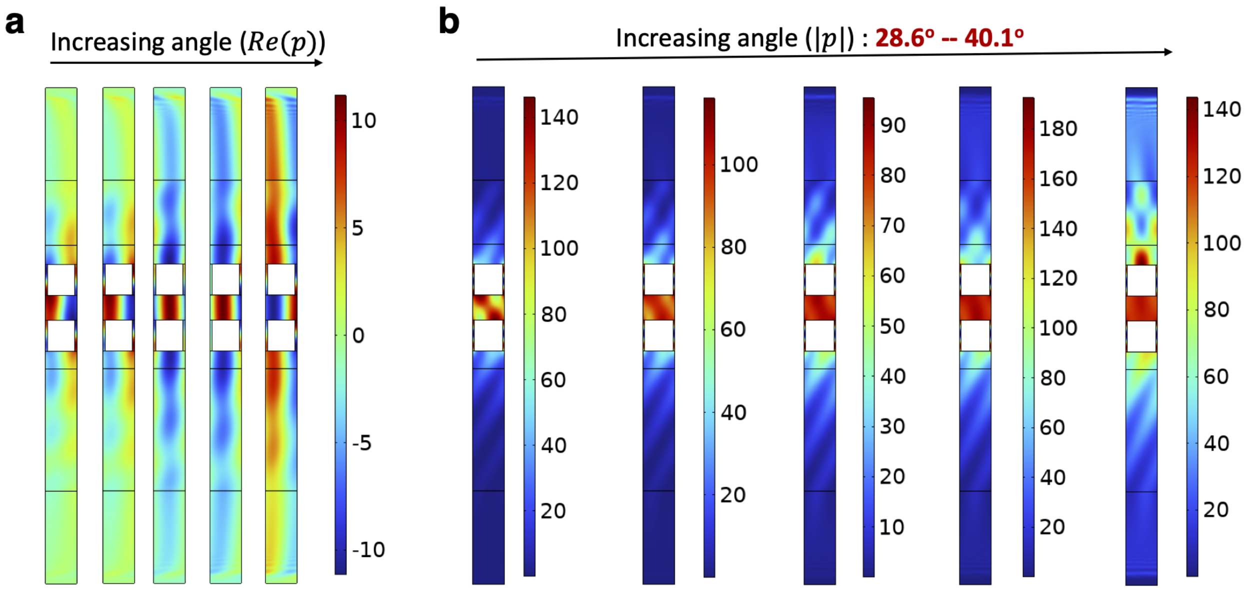

FIG. S3. **Near-fields of the hard-wall material unit-cell. a** Real part and **b** amplitude of the pressure fields for increasing angles at the resonance frequency of the hard-wall structure depicted in Figure S2**b**.

that takes place at 959.4 kHz has only a Q-factor $Q_{HW} = 114.1$, very smaller that Q_{QBIC}. As a hard-wall is an idealistic boundary condition, we choose also a material with ultra-high impedance ($\overline{\eta}_{HI} = 2\times10^3$) and plot the result in Figure S2(a), red-dashed curve. Here also $Q_{HI} = 102.1 \ll Q_{QBIC}$. Figure S2(b) depicts the near-field pressure profile (its real part) at the resonance frequency of around 959 kHz. The white color stands for hard-wall boundary condition. What is evident from the inspection of Figure S2(b) is the leakage of acoustic energy towards the surrounding water, as this resonance can easily couple to free-space scattering, owing to its low Q-factor.

Let us also check the effect of oblique incidence on such meta-atoms made of hard-walls. The results for angles ranging from 28.6° (0.5 rad) to 57.3° (1 rad) are shown in Figure S2(c). Even though an increase of Q_{HW} can be seen, these quality factors are still orders of magnitude smaller than their QBIC counterpart.

The near-field pressure for such structures are shown in Figure S3. What is shown is the strong leakage from these resonant states confirming that the QBIC vanishes if solid silicon is replaced by hard-walls or ultra-high impedance in water.

Fabry-Pérot layers

To conclude this note, we further investigate the effect of closing the slit, i.e., to having a homogeneous (without slits) Fabry-Pérot (FP) layer of silicon, embedded in water. Figures S4(a) and S4(b) show the single and double FP-transmittance, respectively. For the single layer, no resonance is to be observed in the frequency range spanning 0 to 2 MHz, while the double FP

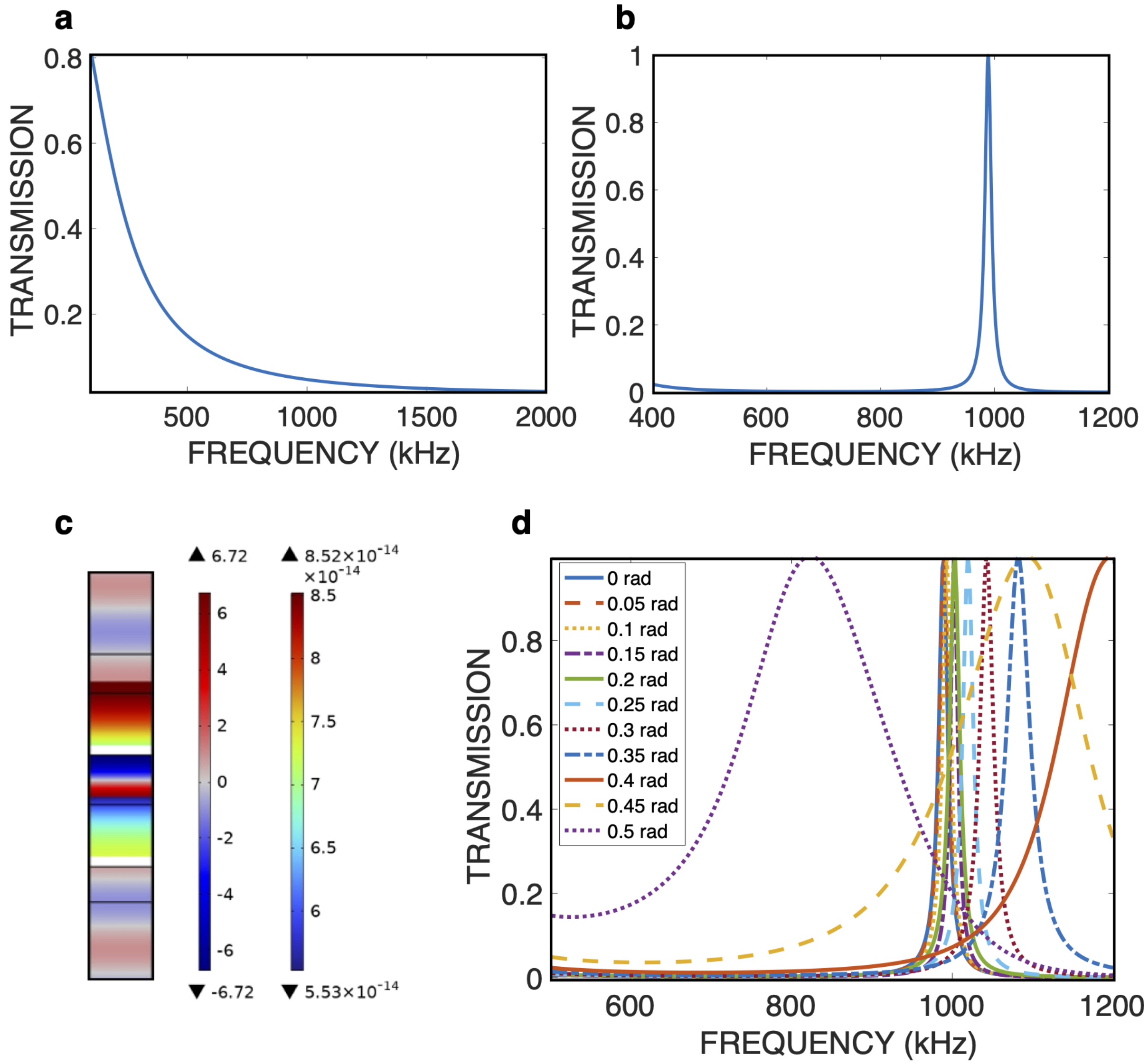

FIG. S4. **Double Fabry-Pérot metasurface.** **a** Transmittance for a single Fabry-Pérot layer made of silicon, embedded in water. **b** Transmittance for the double Fabry-Pérot layers, showing a low-Q resonance at around 1 MHz. **c** Near-field pressure and displacement around the FP resonance of Figure S4**b**. **d** Oblique incidence in the case of a double FP scenario, showing broadband resonances and the absence of QBICs.

shows a resonance at 988.6 kHz. Even if this resonance occurs at similar frequency, as the QBIC in the MS, its Q-factor is much lower (around 72). The near-field shown in the inset, at resonance frequency confirms the leakage of energy as in the case of hard-wall meta-atom. Figure S4(c) shows again the effect of angle of incidence variation, and that no sensible increase of Q_{FP} can be obtained.

The results of this note confirm the importance of having a solid elastic metasurface (with slits) (silicon as we choose here, or other materials, such as Aluminum of steel). The importance of both pressure and shear waves is demonstrated, for the goal of obtaining the ultrasound QBIC.

Different materials

The elasticity of the material composing the metasurface plays a primordial role in the high-Q. To demonstrate this fact, we have performed full-wave simulations where we varied the used material from silicon, steel, iron, and copper. The results are depicted in Fig. S5 and show that silicon offers the best Q-factor among these materials. To understand why, we compute both the pressure and shear acoustic impedance of these materials normalized by that of water, and show them in Table I. What can be seen is that silicon offers the lowest impedance mismatch for both kinds of waves. Steel and iron have a much higher mismatch and thus the Q-factor is lowered, while copper, with a reasonable impedance mismatch, can result in a good Q-factor. Yet, silicon is the best candidate for obtaining the unprecedented Q-factor owing to its elastic properties in aqueous environments, as the other materials behave more like hard-walls, as discussed in the

TABLE I. Table showing the comparison between different materials of the BIC unit-cell.

Material	Q-factor	Pressure impedance	Shear impedance
Silicon	1.6×10^4	15.22	8.41
Steel	360	30.69	16.96
Iron	368	30.73	16.71
Copper	820	29.91	12.93

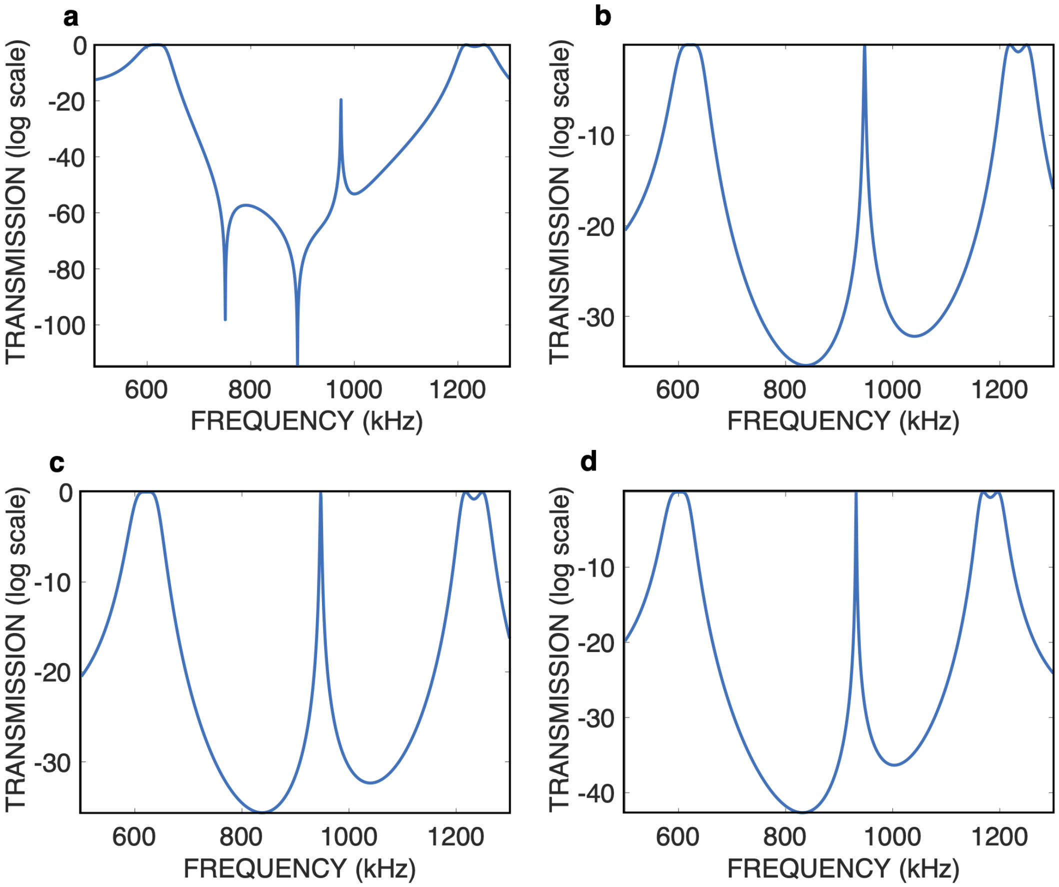

FIG. S5. **Role of material.** Transmittance of BIC metasurface made of **a** silicon, **b** steel, **c** iron, and **d** copper.

sub-section above.

NOTE 3: TUNABILITY OF THE BIC STRUCTURE AND SLIT POSITION

Role of gap on the BIC

The role of the gap between the two metasurfaces g_0 is analyzed herein. We start by plotting in Figure S6(a) (left panel) the QBIC frequency from the numerical simulations (dark-blue squares) and that from the TCMT predictions (light-blue circles) in the left axis. The right axis plots the agreement between these two frequencies denoted f_0 and f_1, respectively and shown in the Table given in Figure S6(b). What can be noticed is that for low gap size as well as large gap size the agreement is moderate. Yet, in the region of interest to our study, i.e., around the gap of 0.8 mm, the agreement is excellent, especially taking into account the intricate acousto-elastic coupling.

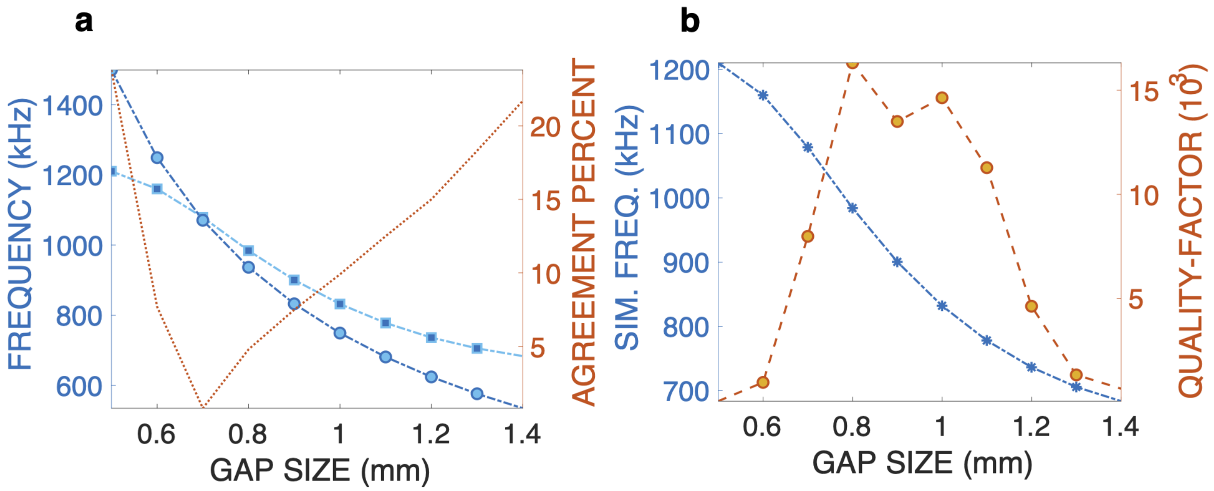

c

g_0 (mm)	f_0 (kHz)	f_1 (kHz)	% Agreement	Q ($\times 10^3$)
0.1	NaN	7493	NaN	No BIC
0.2	NaN	3746.5	NaN	No BIC
0.3	NaN	2497.7	NaN	No BIC
0.4	NaN	1873.3	NaN	No BIC
0.5	1210.2	1498.6	23.8	0.06
0.6	1159.85	1248.8	7.7	0.97
0.7	1078.83	1070.4	0.8	8.07
0.8	984.13	936.6	4.8	16.5
0.9	900.43	832.6	7.5	13.65
1	832.08	749.3	9.9	14.8
1.1	778.06	681.2	12.5	11.4
1.2	736.38	624.4	15	4.67
1.3	705.70	576.4	18.3	1.33
1.4	683.69	535.2	21.7	0.67

FIG. S6. **Role of the gap on the QBIC. a** Frequency of the QBIC from numerical (dark-blue squares) and that from the TCMT predictions (light-blue circles); the agreement is plotted in the right axis. **b** Frequency and Q-factor of the QBIC VS the gap size. **c** Calculations showing the role of the gap on the QBIC.

The right panel of Figure S6(a) gives the frequency and quality-factor of the resonance depending on the gap size. This also shows that the QBIC is mostly localized between gaps of 0.7 mm and 1.1 mm.

Asymmetric slit position

In order to verify the robustness of our design, we need to investigate its sensitivity to geometric parameter variations. This is important, for instance, in experimental validations. First of all, as we have two metasurfaces, with two slits, we allow the lower slit position to change in comparison to the first one, in the x-direction (See inset of Figure S7(a)). The variation ranges from 0 to 0.4 mm. It is evident from Figure S7(a) and its right inset, that asymmetrically positioning the lower slit does not alter the QBIC resonance by any mean. The zoom in the right inset further showcases this observation. Figure S7(b) depicts the pressure field distribution inside the gap and near the metasurfaces and shows that the symmetry of the mode with respect to y-direction is conserved.

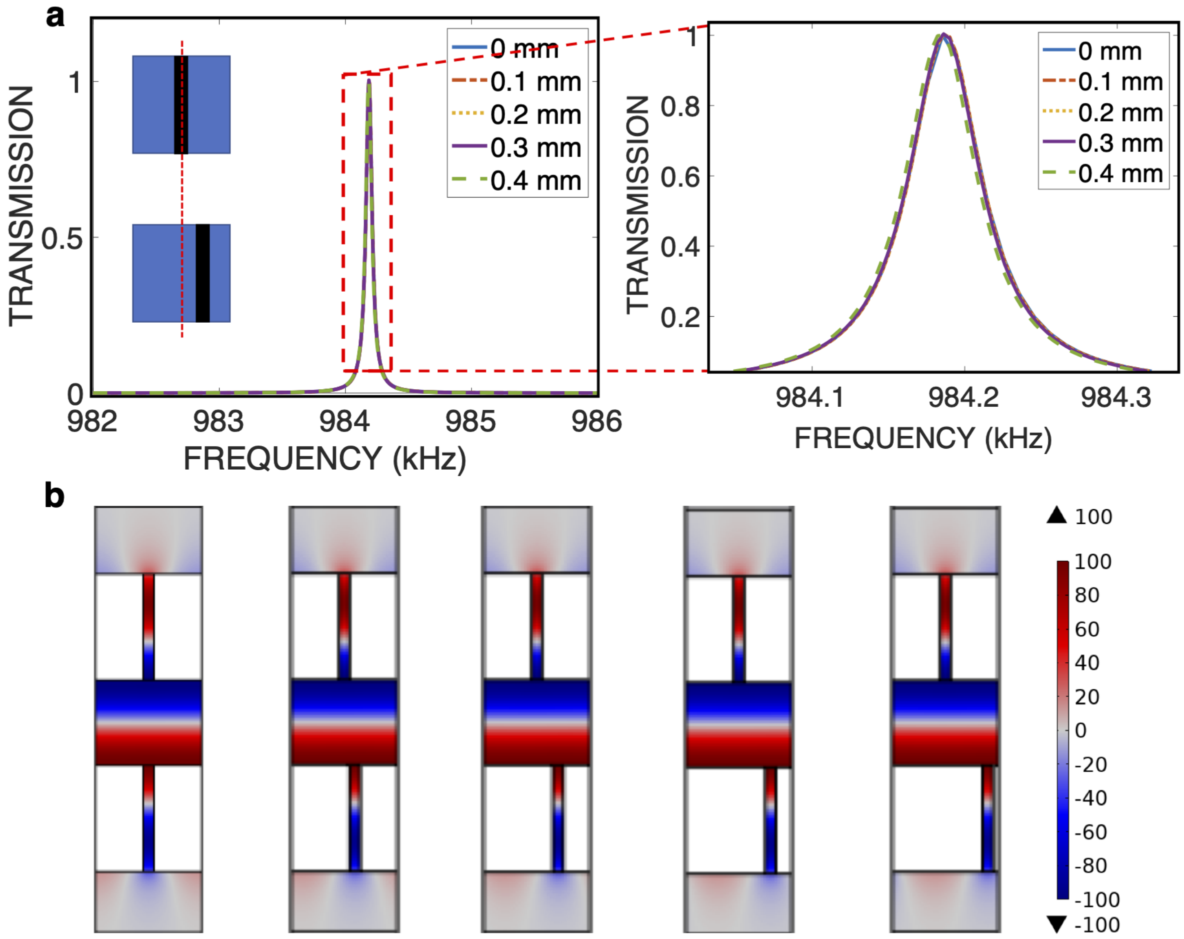

FIG. S7. **Tunability of the BIC resonator structure and slit position effect. a** Transmittance of the QBIC metasurface when the position of the lower slit is varied between 0 mm and 0.4 mm, showing no effect on the resonance. **b** Near-field pressure of the various situations considered in **a**.

This the high localization at the QBIC frequency is conserved.

Role of period and slit size

Let us now turn to the geometric consideration of the period and the slit size. When one of these parameters is considered, the other one is kept constant. We first fix the slit size to 0.1 mm

a

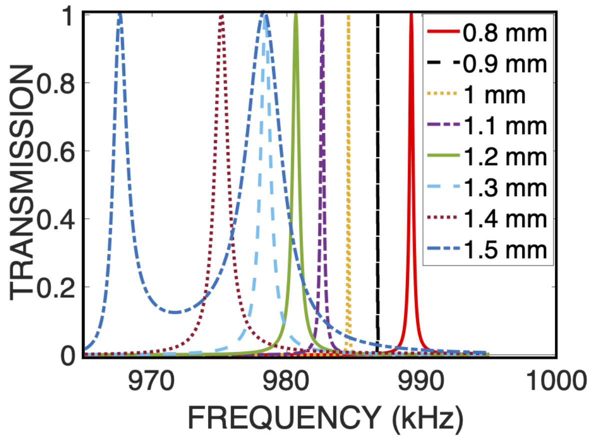

Period (mm)	Freq (kHz)	Q ($\times 10^3$)
0.8	989.218	3.23
0.9	986.730	80.2
1	984.562	16.438
1.1	982.609	3.551
1.2	980.657	1.726
1.3	978.404	1.115
1.4	975.138	0.85
1.5	978.292	0.308

b

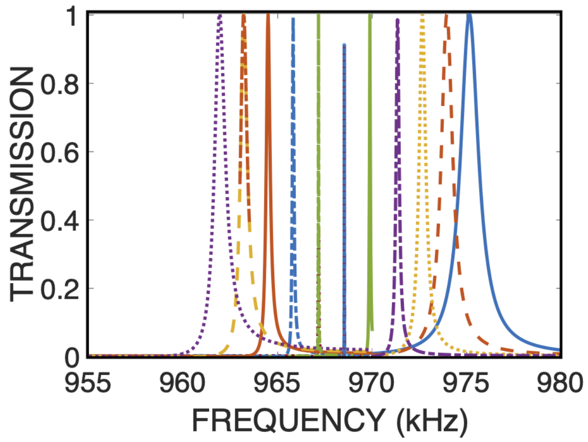

Slit width (mm)	Freq (kHz)	Q ($\times 10^3$)
0.10	975.138	0.863
0.11	973.96	1.334
0.12	972.657	2.329
0.13	971.353	4.856
0.14	969.875	16.922
0.15	968.520	325.146
0.16	967.158	51.36
0.17	965.818	9.676
0.18	964.491	4.043
0.19	963.189	2.228
0.20	961.937	1.431

FIG. S8. **Tunability of the BIC resonator structure and slit position effect.** **a** Transmittance of the QBIC for a fixed slit width and for varying periods, showing a maximum Q-factor of around 80×10^3 for a period of 0.9 mm. **b** Same as in **a** but for a fixed period and for varying slit width, showing a maximum Q-factor of around 325×10^3 for a slit width of 0.15 mm.

and vary the period of the metasurfaces between 0.8 mm and 1.5 mm, and depict the result of the transmittance in Figure S8(a). It is striking to see that for $P = 0.9$ mm, a Q-factor of 80.2×10^3 may be achieved, i.e., almost 5 times the Q-factor of $P = 1$ mm. Then, for increasing P, the Q is deteriorated, and falls to 0.3×10^3 for $P = 1.5$ mm.

Now, if we fix the period $P = 1$ mm and vary the slit width w_0, instead from 0.1 to 0.2 mm. we can see in Figure S8(b) an even more dramatic increase of the Q-factor. Namely, for $w_0 = 0.15$ mm, the maximum Q can be obtained and reaches 325×10^3. Then, for increasing or decreasing slit widths the Q drops substantially, below 10^3.

Figure S9(a) shows the effect of the slit change as well as the resonance frequency shift.

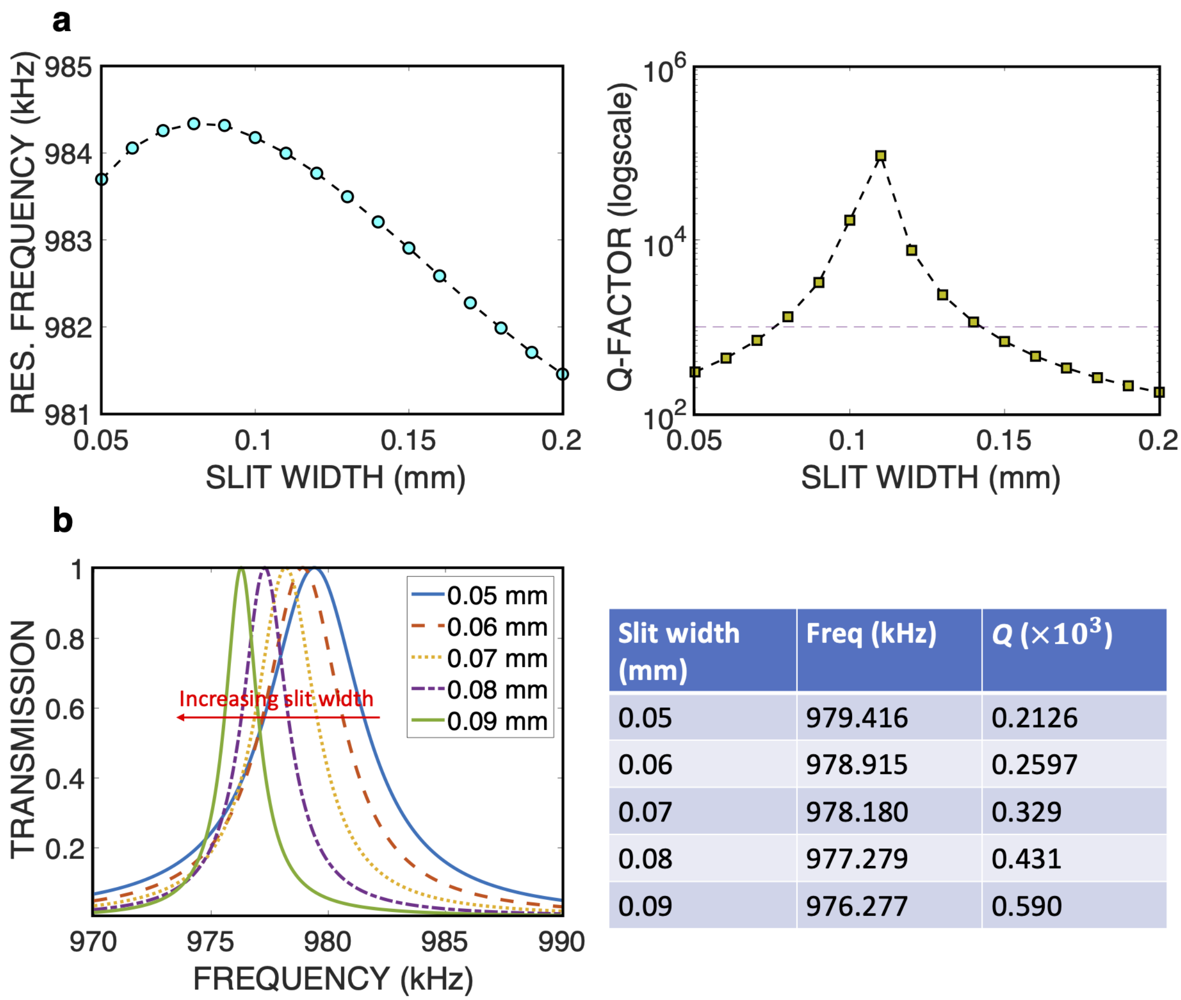

Slit width (mm)	Freq (kHz)	Q ($\times 10^3$)
0.05	979.416	0.2126
0.06	978.915	0.2597
0.07	978.180	0.329
0.08	977.279	0.431
0.09	976.277	0.590

FIG. S9. **Tunability of the BIC resonator structure and slit position effect.** **a** Resonant frequency and Q-factor for the QBIC with varying slit width. **b** Effect on transmittance of a small slit width variation, showing the deterioration of the QBIC.

Figure S9(b) depicts the the effect of having a small slit width, and counter-intuitively, a small slit size results in a very low Q-factor that does not qualify to a QBIC.

Role of gap and periodicity on oblique incidence

In this sub-section we emphasize the primordial role of elastic waves (both longitudinal and transverse) in the generation of the BIC effect. For instance, we have shown in our work, that using the same geometry but with a hard-wall boundary condition instead of silicon, as shown in Figure S2 does not lead in fact to the ultra-high Q-factor. This undoubtedly shows the role of the interplay between solid and fluid interactions that enables the elastic BIC and the subsequent high Q-factor predicted theoretically and further observed experimentally. Moreover the maximum Q-factor observed here at 10 degrees results from a complex dependency of all the parameters of the device, such as periodicity, gap thickness, material composition, slit width, etc. To demonstrate this, we perform additional full-wave simulations where we change separately the gap size (and keeping everything else similar to the result of Figure XX of the MS) and the periodicity (by keeping the other parameters same, as before). The results are shown in Fig. S10. It can be seen that a slight increase of the gap results in a maximum of Q-factor observed now at 13 degrees (Fig. S10(a)), while an increase of period from 1 mm to 1.2 mm decreases the maximum angle to

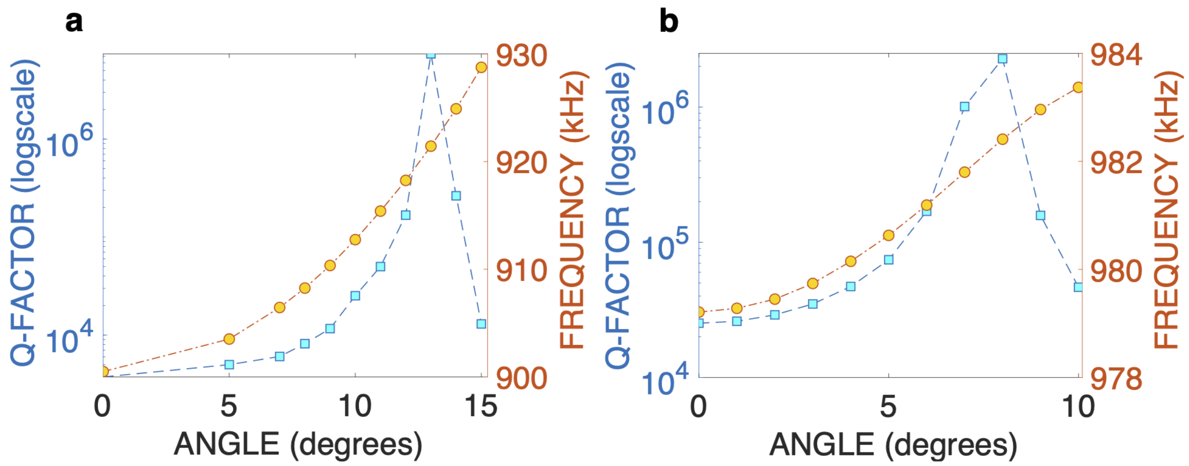

FIG. S10. **Role of gap size and periodicity on the oblique incidence.** Plot of the Q-factor in logarithmic scale (left axis) and resonant frequency (right axis) for the same metasurface of the manuscript but with **a** a gap of 0.9 mm and **b** a period of 1.2 mm.

8 degrees (Fig. S10(b)).

Coupling VS the gap

Figure S11 depicts the variation of the coupling coefficient $\delta/2\pi$, given in Eq. (1) of the manuscript, VS the gap size g_0 in mm. δ is computed by comparing the frequency of the single resonator to the frequency of the QBIC. It can be seen from Figure S11 that δ increases in a logarithmic fashion with g_0.

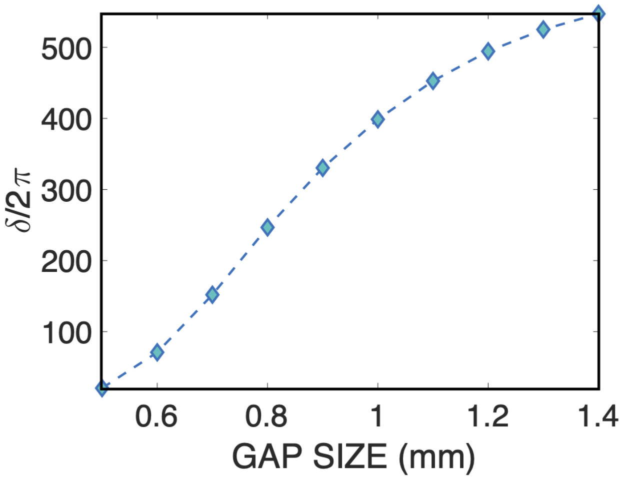

FIG. S11. **Coupling coefficient VS the gap.** Variation of $\delta/2\pi$ VS the grap size in mm.

NOTE 4: EXPERIMENTAL DETAILS AND ROLE OF THE VISCOSITY

As we are working at high frequencies (a few MHz) in aqueous environment, and as we have in our meta-atoms, small slits of 0.1 mm or even smaller, it is important to consider the role of viscosity on our results. Figure S12(a) gives the viscosity of water as function of temperature (both dynamic and kinematic) [2]. In our numerical simulations, we considered a non-viscous fluid, where there is no internal friction, i.e., no energy dissipation. We show in the following that this approximation is valid. To get an order of magnitude of the effect, we consider harmonic variation with time, thus we make the substitutions: $\partial/\partial t \to -i\omega$ and $\partial/\partial \mathbf{k} \to i\mathbf{k}$.

The first acoustic equation, i.e., mass conservation is unchanged, if we consider viscosity. The second equation, i.e., momentum conservation is re-written as [3]

$$-i\omega \mathbf{v}' + \mathbf{k}\left[\frac{\rho'}{\rho_0} - \left(\nu' - \nu\right)\mathbf{k}\cdot\mathbf{v}'\right] - \nu \mathbf{k}^2 \mathbf{v}', \quad (8)$$

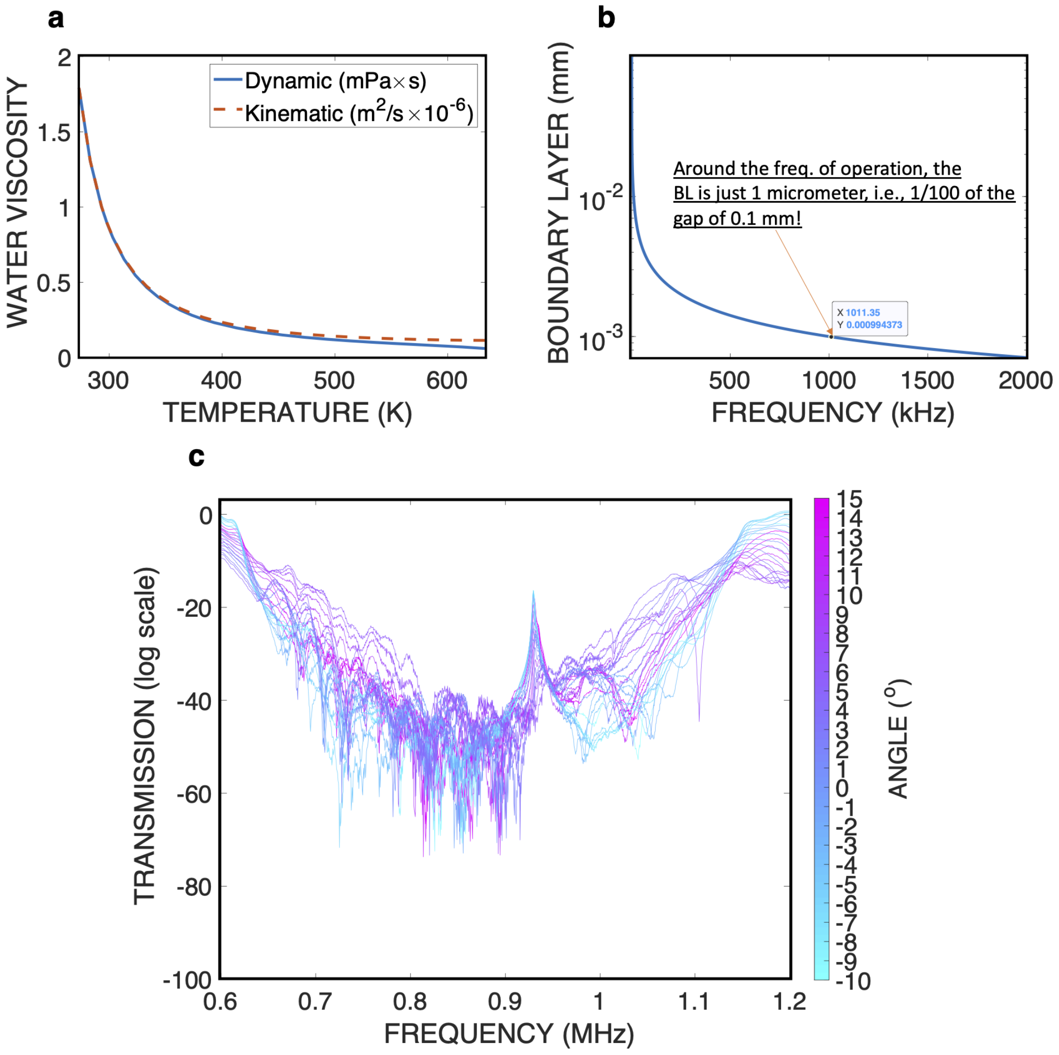

FIG. S12. **Role of viscosity and experimental results.** **a** Variation of the dynamic (solid-blue line) and kinematic (red-dashed line) viscosity of water with temperature. **b** Boundary layer thickness VS frequency for water. **c** Magnitude of the transmittance in logarithmic scale when oblique incidence of various angles is measured experimentally.

the last two terms of which are solely due to viscosity.

Then, let us compare $\omega|\mathbf{v}'|$ and $\nu\mathbf{k}^2|\mathbf{v}'|$, i.e., ω and $\nu\mathbf{k}^2$; thus

$$\frac{\omega}{\nu\mathbf{k}^2} = \frac{c^2\omega}{\nu\omega^2} = \frac{c^2}{2\pi\nu f}\,. \tag{9}$$

If we consider $c = 1480$ m/s, $f = 10^6$ Hz, $\nu = 10^{-6}$ m^2/s; then

$$\frac{\omega}{\nu\mathbf{k}^2} = 3.5\times 10^5 \gg 1\,. \tag{10}$$

Hence, the term due to viscosity, in Eq. (8) is much smaller than the other terms. The effect due to viscosity can hence be neglected for ultrasound propagation in water.

We can also define a 'boundary layer' δ where effects of viscosity can be felt, and beyond which these could be ignored, i.e., $\delta = \sqrt{\nu t}$, where ν is the kinematic viscosity and t is time scale. t can be replaced by $2\pi/\omega$, for orders of magnitude; hence $\delta = \sqrt{2\pi\nu/\omega}$. δ is plotted VS frequency between 0 and 2 MHz in Figure S12(b). For frequencies of the order of 1 MHz, $\delta \approx 10^{-3}$ mm. As our slits are of the order of 10^{-1} mm, this means that the boundary layer is 2 orders of magnitude smaller, which means its effects are very small to be detected in experimental setups.

To conclude this note, we give the experimental results of oblique incidence on the QBIC Q-factor in Figure S12(c).

NOTE 5: ROBUSTNESS OF THE BIC EFFECT WITH PARAMETER VARIATION

Long-wavelength limit

In order to compare our findings to some studies that considered the response of similar structures but using a single slit [4], we show the effect of increasing the period of our metasurface while keeping the slit size constant. The period that we use in the MS is 1 mm and the slit size is

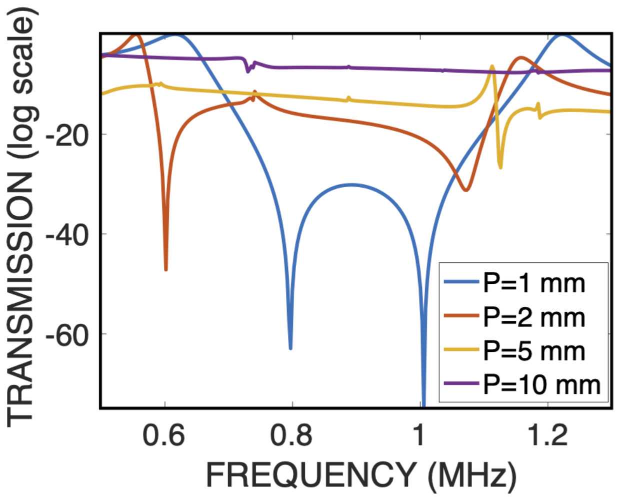

FIG. S13. **Effect of periodicity.** Effect of increasing the period on the transmittance of the single metasurface of Fig. 2a of the MS.

0.1 mm. We choose now periods of 2 mm, 5 mm, and 10 mm. When the period is as big (100 times the slit size for the last scenario) then the periodicity effect will be minimal. What can be seen from Figure S13 is that when the period increases the transmittance decreases and for a period of 5 or 10 mm, the metasurface becomes mostly reflective. This is anticipated as the slit is too narrow and the solid metasurface will act as a mirror for sound waves at these frequencies, in our case.

Parameter variation change

To further analyze the robustness of our BIC device, i.e., on as to what extent the parameters of the material change influence the BIC, and the possibility of their prominent impact on the results, we perform additional simulations where we change the temperature of the fluid and investigate the robustness of the BIC with this regard. The result is shown in Figure S14a and it clearly shows a blueshift with increasing temperature. Yet, the BIC is robust and persists at any temperature.
Next, the variation of transmission when the loss in the slits is varied is also shown in Figure S14a, in a more complete way and complements thus the results of Figure 4 of the MS.
In fact, what we have seen in this study is that the BIC is very sensitive to material parameter variation as well as to geometric variation. Yet, the effect is robust enough and this explains the experimental observation of such unprecedented Q-factors.

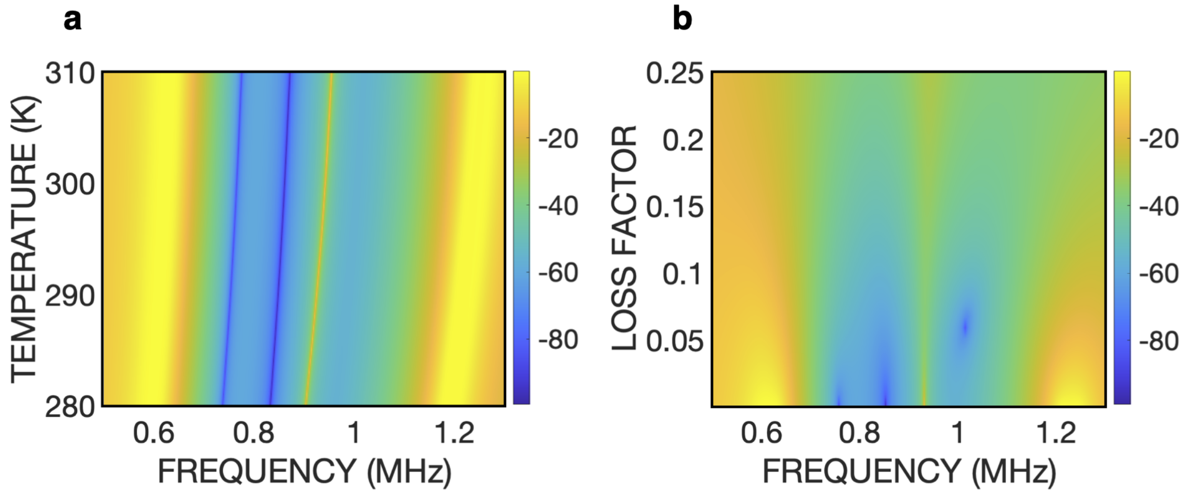

FIG. S14. **Effect of temperature and loss. a** Transmission in logarithmic scale for the BIC system VS frequency in MHz and temperature of the water in K. **b** Transmission in logarithmic scale for the BIC system VS frequency in MHz and the loss factor in the water inside the slits.

Steel and silicon cavity-resonators

In order to build a state-of-the-art phononic crystal cavity resonator that is supposed to have the highest Q-factor, we use a finite-sized crystal with a defect at its center, via two configurations made of steel and silicon. To justify our choice for steel as defect-cavity resonator, we show here in Figure S15 the comparison between the two cavities of exactly the same geometries, and at the same frequencies with the notable difference of using either steel or silicon as material of the phononic rods. The results demonstrate that steel offers a better Q-factor. Hence, we build experimentally a phononic crystal made of steel and we compare it to the BIC results in the MS.

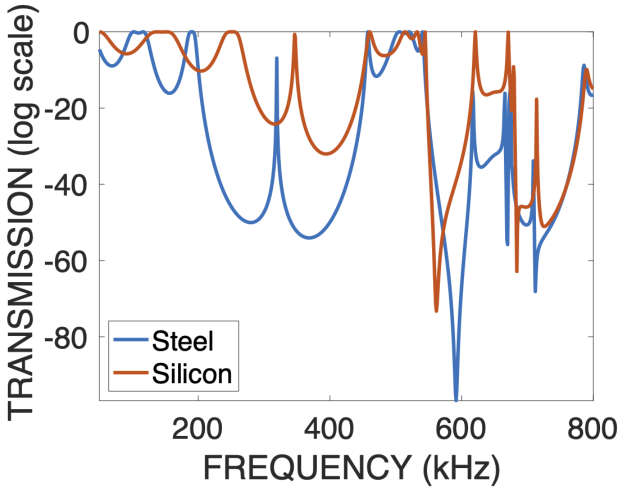

FIG. S15. **Phononic cavity.** Transmission in logarithmic scale for the phononic crystal cavity made of steel (blue-solid line) and silicon (red-solid line).

NOTE 6: COMPARISON OF Q-FACTOR WITH STATE-OF-THE-ART

As mentioned in our manuscript, the quest for high Q-factors in acoustics is an important endeavor. The use of acoustic BIC is one of the most promising avenues to realize this objective. Very recently, some designs were proposed to build high-Q devices for acoustic, such as the works of Huang *et al.* including sound trapping in open resonators and topological supercavities with Q-factors of around 400 at around 3 kHz [5–7]. Liu *et al.* proposed Friedrich-Wintgen BIC by

TABLE II. Table showing the comparison of Q-factors of state-of-the-art acoustic devices with respect to our own contribution.

Reference	Q-factor	Frequency	Environment	Other notes
[5]	≈ 400	≈ 3 kHz	Airborne	
[12]	Few tens	≈ 1 MHz	**Waterborne**	(Q is not given but deduced)
[6]	≈ 340	≈ 3 kHz and ≈ 5 kHz	Airborne	
[7]	NA	≈ 3 kHz	Airborne	(Q is not given)
[8]	≈ 50	≈ 1 kHz	Airborne	
[9]	≈ 180	≈ 5 kHz	Airborne	
[10]	≈ 320	≈ 2 kHz	Airborne	
[11]	≈ 400	≈ 4 kHz	Airborne	
Our Study	≈ 350	≈ 1 MHz	**Waterborne**	

leveraging the near-field coupling to obtain a Q of around 50 at 1 kHz [8], whereas Marti-Sabate *et al.* investigated acoustic accidental BIC at 5 kHz with a maximum Q of 180 [9]. Kronowetter *et al.* considered a scheme similar to that of Huang *et al.* to obtain a BIC at 2 kHz with a maximum Q of 320 in air [10]. Last but not least, Jia *et al.* proposed symmetry-protected BIC in a 3D configuration to get an acoustic resonator at 4 kHz with a maximum Q of around 400, also in air [11], as is the case of all the previously cited examples.

The BIC for underwater ultrasound are almost nonexistent. The study of Amrani *et al.* used continuous elastic layers and resulted in measured Q-factors of the order of a few tens [12]. Our study, to the best of our knowledge, proposes the highest Q-factor for underwater ultrasound.

In fact, our resonator has a Q-factor that is comparable to state-of-the-art devices that were performed at lower frequecnies and in an airborne configuration.

Hence, we believe that our design represents a significant advancement in the domain of ultrasound underwater resonators.

[1] K. F. Graff, *Wave motion in elastic solids* (Courier Corporation, 2012).

[2] L. Korson, W. Drost-Hansen, and F. J. Millero, Viscosity of water at various temperatures, The Journal

of Physical Chemistry **73**, 34 (1969).

[3] D. Homentcovschi and R. N. Miles, Influence of viscosity on the reflection and transmission of an acoustic wave by a periodic array of screens: The general 3-d problem, Wave Motion **45**, 191 (2008).

[4] A. Bozhko, V. M. García-Chocano, J. Sánchez-Dehesa, and A. Krokhin, Redirection of sound in straight fluid channel with elastic boundaries, Physical Review B **91**, 094303 (2015).

[5] L. Huang, Y. K. Chiang, S. Huang, C. Shen, F. Deng, Y. Cheng, B. Jia, Y. Li, D. A. Powell, and A. E. Miroshnichenko, Sound trapping in an open resonator, Nature communications **12**, 4819 (2021).

[6] L. Huang, B. Jia, A. S. Pilipchuk, Y. Chiang, S. Huang, J. Li, C. Shen, E. N. Bulgakov, F. Deng, D. A. Powell, *et al.*, General framework of bound states in the continuum in an open acoustic resonator, Physical Review Applied **18**, 054021 (2022).

[7] L. Huang, B. Jia, Y. K. Chiang, S. Huang, C. Shen, F. Deng, T. Yang, D. A. Powell, Y. Li, and A. E. Miroshnichenko, Topological supercavity resonances in the finite system, Advanced Science **9**, 2200257 (2022).

[8] S. Liu, S. Huang, Z. Zhou, P. Qian, B. Jia, H. Ding, N. Wang, Y. Li, and J. Chen, Observation of acoustic friedrich-wintgen bound state in the continuum with bridging near-field coupling, Physical Review Applied **20**, 044075 (2023).

[9] M. Martí-Sabaté, J. Li, B. Djafari-Rouhani, S. A. Cummer, and D. Torrent, Observation of two-dimensional acoustic bound states in the continuum, Communications Physics **7**, 122 (2024).

[10] F. Kronowetter, M. Maeder, Y. K. Chiang, L. Huang, J. D. Schmid, S. Oberst, D. A. Powell, and S. Marburg, Realistic prediction and engineering of high-q modes to implement stable fano resonances in acoustic devices, Nature Communications **14**, 6847 (2023).

[11] B. Jia, L. Huang, A. S. Pilipchuk, S. Huang, C. Shen, A. F. Sadreev, Y. Li, and A. E. Miroshnichenko, Bound states in the continuum protected by reduced symmetry of three-dimensional open acoustic resonators, Physical review applied **19**, 054001 (2023).

[12] M. Amrani, I. Quotane, C. Ghouila-Houri, L. Krutyansky, B. Piwakowski, P. Pernod, A. Talbi, B. Djafari-Rouhani, *et al.*, Experimental evidence of the existence of bound states in the continuum and fano resonances in solid-liquid layered media, Physical Review Applied **15**, 054046 (2021).
